# Determinants of the Seasonal Influenza Vaccination Uptake Among People Aged 50 Years or Above During and After the Pandemic: A Systematic Review

**DOI:** 10.3390/vaccines14080705

**Published:** 2026-08-15

**Authors:** Liwen Ding, Siyu Chen, Fuk-yuen Yu, Ka Hei Chan, Yuan Fang, Phoenix K. H. Mo, Zixin Wang

**Affiliations:** Centre for Health Behaviours Research, JC School of Public Health and Primary Care, The Chinese University of Hong Kong, Hong Kong, China

**Keywords:** seasonal influenza vaccination, COVID-19, determinants, older adults, middle-aged adults

## Abstract

**Background/Objectives:** Seasonal influenza vaccination (SIV) coverage remains suboptimal among adults aged ≥50 years, and the COVID-19 pandemic might influence the determinants of SIV uptake. This systematic review aimed to identify determinants of SIV uptake among adults aged ≥50 years during and after the COVID-19 pandemic. **Methods:** We searched PubMed, MEDLINE, Embase, Web of Science, Global Health, CINAHL, Cochrane Library, APA PsycINFO, and APA PsycArticles for studies published between 1 December 2019 and 1 January 2026. The pooled prevalence of SIV uptake was synthesized using random-effects models in meta-analysis. **Results:** A total of 50 studies were included in the systematic review. COVID-19-related factors, such as perceived risk of co-infection with SARS-CoV-2 and seasonal influenza and concerns about potential interactions between COVID-19 vaccines and SIV, were determinants of SIV uptake. Other modifiable determinants included those that existed before the pandemic (e.g., knowledge and perceptions of seasonal influenza and SIV). Subgroup analysis showed that cost and inaccessibility were barriers to receiving SIV in places without a fully subsidized SIV, and perceived susceptibility and vaccine safety concerns were commonly identified as facilitators and barriers in studies with data collected after the pandemic. The pooled prevalence of SIV uptake among adults aged 50 years or above was 53% (95% confidence interval: 44–62%). Such uptake appeared lower among adults aged 50–64 years, in regions without a fully subsidized SIV, and after the COVID-19 pandemic. **Conclusions:** These findings could inform service planning and interventions promoting SIV uptake among adults aged ≥50 years in the post-pandemic era. More studies are needed to explore determinants specific to people aged 50–64 years to inform health promotion tailored to their needs.

## 1. Introduction

Seasonal influenza remains a major global health threat, causing approximately 1 billion infections, 3–5 million cases of severe illness, and 0.29–0.65 million respiratory-related deaths each year [1]. Adults aged 50 years or above are at increased risk of influenza-related complications, hospitalization, and death [2,3,4]. Adults aged 50–64 years, in particular, constitute an increasingly important risk group, as the burden of chronic conditions rises substantially in this age range while continued workforce participation sustains frequent occupational and social exposure to influenza [5,6]. The emergence of COVID-19 has further complicated the prevention and control of respiratory infections. During the pandemic, co-infection with seasonal influenza and COVID-19 has been associated with an increased risk of hospitalization and death [7,8]. After the pandemic, the relaxation of COVID-19 control measures may have increased population susceptibility to seasonal influenza infection [9,10].

Seasonal influenza vaccination (SIV) is an effective and safe strategy for reducing influenza-related morbidity and mortality among adults aged 50 years or above [11]. Evidence suggests that SIV may not only offer benefits in reducing severe cases among people aged 65 years and older, but also provide greater benefits in reducing influenza-related hospitalizations among people aged 50 to 64 years [12,13]. The World Health Organization (WHO) recommends that older adults (e.g., aged 65 years or above) should be prioritized for SIV as a key group [14], and around 28% of countries worldwide have incorporated this priority recommendation into their national immunization programs [15]. In recent years, some regions, such as some European Union countries and Hong Kong, have extended the recommended age for SIV eligibility to include adults aged 50 years or above after the COVID-19 pandemic due to the increasing risk among those aged 50–64 years [16,17,18].

Despite these recommendations, SIV coverage remains suboptimal. Globally, the median SIV coverage among older adults was only 55% in 2022, which remains below the WHO target of 75% coverage [19]. Coverage is even lower among adults aged 50–64 years in settings where fully subsidized SIV programs are unavailable, with reported uptake ranging from 10% to 32% [20,21,22]. This suboptimal coverage highlights the need to better understand the determinants of SIV uptake among adults aged 50 years or above.

Several systematic reviews have examined determinants of SIV uptake among adults aged 50 years or above, but most included studies were conducted before the COVID-19 pandemic [23,24,25]. The determinants of SIV uptake may have changed during and after the pandemic. On the one hand, the COVID-19 pandemic heightened perceived risks of respiratory infections [26], concerns about co-infections with influenza [27], and increased awareness of vaccine importance [28], which were associated with greater SIV intention or uptake. On the other hand, COVID-19-related disruptions to routine healthcare [29], vaccine fatigue [30], concerns about vaccine safety [31], and worries about possible interactions between COVID-19 vaccination and SIV [27] may have created new barriers to SIV uptake. Two systematic reviews have included studies conducted after the pandemic [32,33]. However, these reviews mainly focused on older adults, rather than the broader population aged 50 years or above, despite evidence that adults aged 50–64 years often have lower SIV uptake. In addition, previous reviews have examined a limited range of determinants and have not sufficiently summarized COVID-19-related factors that may influence SIV uptake during and after the pandemic. A comprehensive synthesis focusing on adults aged 50 years or above is therefore needed to inform targeted strategies for improving SIV coverage.

To address these gaps, this systematic review aimed to identify the determinants of SIV uptake among adults aged 50 years or above during and after the pandemic. It also aimed to synthesize the reported levels of SIV uptake in this population.

## 2. Materials and Methods

This systematic review was registered with PROSPERO (CRD420261298587) and conducted according to the PRISMA (Preferred Reporting Items for Systematic Reviews and Meta-Analyses) guidelines.

### 2.1. Search Strategy

We searched PubMed, MEDLINE, Embase, Web of Science, Global Health, CINAHL, Cochrane Library, APA PsycINFO, and APA PsycArticles for studies published between 1 December 2019 and 1 January 2026. The search was restricted to studies published after December 2019 to capture determinants of SIV uptake during and after the pandemic, given that the pandemic substantially changed vaccination perceptions, policies, and healthcare access [34]. The strategy combined relevant terms and phrases for influenza, vaccination, age group of 50 years or above, and determinants, linked using Boolean operators “OR” and/or “AND”. The detailed search strategies are presented in Table 1 and Supplement S1.

### 2.2. Inclusion and Exclusion Criteria

The eligible studies were selected based on the PEO (participants, exposure, and outcome) criteria. Specifically, P = adults aged 50 years or above or studies reporting results among subgroups of participants in this age group; E = qualitative determinants and/or quantitative reasons related to SIV uptake or intention; O = quantitative measures and/or qualitative findings of SIV uptake and intention. Population-based observational studies (including quantitative, qualitative, and mixed-methods studies) published in English and conducted in any country or setting were included. Table 2 shows the full inclusion and exclusion criteria for our study.

### 2.3. Data Extraction

Information from quantitative, qualitative, and mixed-methods studies was extracted by two independent reviewers (LD and SC) in parallel, and disagreements regarding the extraction, categorization, or interpretation of findings were resolved through discussion with the senior reviewer (ZW). The extracted information included: study characteristics (e.g., first author, publication year, country, region, policy context, study design), participant information (e.g., sample size, age group), study outcomes (e.g., SIV uptake/intention), and main findings (e.g., prevalence of SIV uptake/intention, facilitators, barriers).

For quantitative studies and the quantitative components of mixed-methods studies, we extracted the prevalence of SIV uptake or intention and the reported facilitators and barriers associated with SIV uptake or intention. For qualitative studies and the qualitative components of mixed-methods studies, we extracted participant-reported reasons related to SIV uptake or intention. Relevant participant-reported reasons, facilitators, and barriers from both quantitative and qualitative evidence were grouped into broader determinant domains, including knowledge and attitudes, socio-demographics, COVID-19-related factors, and social influence.

### 2.4. Quality Assessment

The Mixed Methods Appraisal Tool (MMAT) version 2018 was used to assess the quality of the included studies, which allows critical appraisal across quantitative, qualitative, and mixed-methods studies in one framework [35]. In addition to the two initial screening questions, methodological quality was assessed using the relevant MMAT criteria according to study design. For quantitative studies, quality was assessed according to five criteria: whether the sample was representative of the target population, whether the measurements were appropriate, whether outcome data were complete, whether potential confounders were adequately considered where applicable, and whether the statistical analyses were appropriate to answer the research question. For qualitative studies, validity was evaluated according to five criteria: whether the qualitative approach was appropriate for the research question, whether the data-collection methods were adequate, whether the findings were adequately derived from the data, whether the interpretation of the results was sufficiently supported by the data, and whether there was coherence among the data sources, data collection, analysis, and interpretation. For mixed-methods studies, both the relevant quantitative and qualitative components and the integration of these components were assessed using the corresponding MMAT criteria. Each criterion was rated as “Yes”, “No”, or “Can’t tell”, with the number of criteria met out of five reported. These quality assessments were also conducted by two reviewers (LD and SC) simultaneously, with any discrepancies resolved through discussion with the senior reviewer (ZW).

### 2.5. Data Synthesis and Meta-Analysis

Qualitative findings from qualitative studies and the qualitative components of mixed-methods studies were synthesized narratively, and areas of convergence or divergence between the qualitative and quantitative evidence were identified. The qualitative findings were not included in the meta-analysis because they were not expressed as comparable quantitative effect estimates.

For quantitative studies and the quantitative components of mixed-methods studies, pooled prevalence estimates of SIV uptake and SIV intention were synthesized using random-effects models in meta-analysis. Heterogeneity was assessed using the *I*^2^ statistic [36,37], and funnel plots and Egger’s tests were used to evaluate potential publication bias [37]. Trim-and-fill estimation was performed if significant publication bias was detected [38]. Subgroup analyses were conducted by age group (50–64 vs. 65+), geographic regions defined by the WHO (African Region, Region of the Americas, South-East Asia Region, European Region, Eastern Mediterranean Region, and Western Pacific Region), vaccination policy (fully subsidized vs. self-financed/partially subsidized), and vaccination season (during the COVID-19 pandemic vs. after the COVID-19 pandemic). The World Health Organization declared the end of the COVID-19 pandemic on 5 May 2023 [39]. Therefore, we performed subgroup analyses according to whether the data were collected before or after 5 May 2023. All statistical analyses were performed via RevMan 5.4.1 (The Cochrane Collaboration, Software Update, Oxford, UK) and R (version 4.2.3; R Foundation for Statistical Computing, Vienna, Austria) with the “*metafor*” package. A two-tailed *p*-value < 0.05 was considered statistically significant.

## 3. Results

### 3.1. Identification of Studies

**Figure 1:**
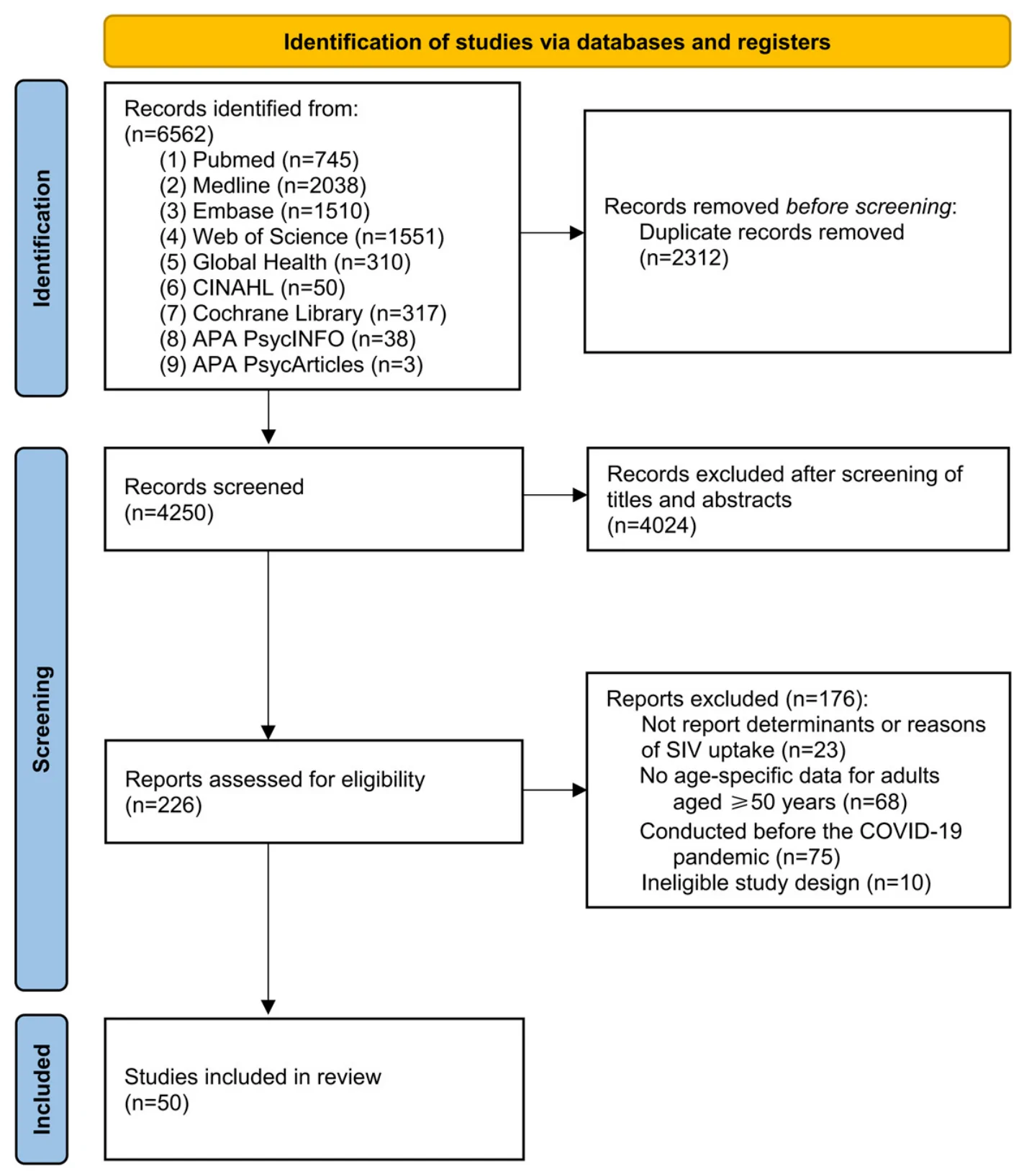
Flowchart of study selection. SIV—seasonal influenza vaccination.

A total of 6562 studies were initially identified. After excluding 2312 duplicates and 4024 ineligible studies after screening the titles and abstracts, 226 studies were selected for full-text review. Among these, 176 studies were excluded for the following reasons: they did not report determinants or reasons for SIV uptake (n = 23), they had no age-specific data for adults aged ≥50 years (n = 68), they were conducted before the COVID-19 pandemic (n = 75), and they had an ineligible study design (n = 10). Ultimately, 50 studies were included in this systematic review (Figure 1).

### 3.2. Overview of Included Studies

The characteristics of the included studies are shown in Table 3. Regarding the study outcomes of the included studies, 37 reported SIV uptake [26,27,31,40,41,42,43,44,45,46,47,48,49,50,51,52,53,54,55,56,57,58,59,60,61,62,63,64,65,66,67,68,69,70,71,72,73], seven reported SIV intention [74,75,76,77,78,79,80], and six reported both [22,81,82,83,84,85]. The data were collected from February 2020 to January 2025, with 31 studies collecting data during the COVID-19 pandemic, 18 studies collecting data after the pandemic ended, and one study covering both periods. For study design, the included studies comprised 45 cross-sectional studies [22,26,27,40,41,42,43,44,45,46,47,48,49,50,52,53,54,55,56,57,58,59,60,61,62,64,65,66,67,68,69,70,71,72,75,76,77,78,79,80,81,82,83,84,85], two qualitative studies [63,73], and three mixed-methods studies [31,51,74]. Regarding study regions, 13 studies were conducted in the Americas region [26,31,41,42,46,47,48,54,55,62,70,72,74], four in the Eastern Mediterranean region [43,61,69,71], nine in the European region [40,44,45,49,50,53,57,63,64], and 24 in the Western Pacific region [22,27,51,52,56,58,59,60,65,66,67,68,73,75,76,77,78,79,80,81,82,83,84,85]. Among these, excluding some unreported cases, 22 study regions implemented a fully subsidized SIV [27,31,40,41,42,43,47,48,49,50,52,53,54,55,63,64,70,75,76,80,83,85], while 16 study regions were self-financed or partially subsidized [22,45,51,56,58,60,61,67,69,73,77,78,79,81,82,83]. The included studies involved 4,605,615 adults aged 50 years or above. Six studies provided specific data for people aged 50–64 years [26,62,63,67,70,74], and 21 studies reported specific data for those aged 65 years and older [26,27,31,40,42,43,47,50,51,53,54,55,61,62,64,66,68,70,72,75,76]. The remaining studies covered both age groups or only reported the mean age of the participants. In the quality assessment, all studies were found to have clear research questions and collected data that allowed them to address these questions. The number of criteria met by the included studies according to the MMAT ranged from 4 to 5 out of 5 (Supplement S2).

### 3.3. Quantitative Findings

#### 3.3.1. Socio-Demographics

Higher income [26,42,45,46,50,52,58,60,61,62,65,71,72,82,84,85] and higher education [26,54,58,61,62,64,65,71,72,82] were identified as factors associated with greater SIV uptake in most studies. Eleven studies found that older age was associated with higher SIV uptake [26,27,46,48,49,52,54,59,62,82,85], whereas one study suggested that younger age was associated with higher uptake [60]. Being married [46,65,67,71,85], being a local resident [22,59,85], having health insurance coverage [47,57,59,61,65,85], and residing in nursing homes [82] were identified as facilitators of SIV uptake, whereas being male [22,27,46,59,82] and being employed [27,46,61,71,85] were reported as barriers. With the exception of one study [61], four studies indicated that living in urban areas was also a facilitator of SIV uptake [59,62,64,82]. Evidence regarding living with family was mixed: two studies identified it as a facilitator of SIV uptake [58,64], while four studies considered it a barrier [27,46,50,51] (Table 4).

#### 3.3.2. Health Conditions

Eleven studies indicated that middle-aged and older adults with chronic diseases, such as cardiovascular disease [49], COPD [65] and hyperlipidemia [65], were more likely to receive SIV [22,26,27,47,49,50,57,58,65,67,71]. A history of vaccine-preventable diseases was also identified as a factor promoting SIV uptake [22,59,60,61,85] by most studies, particularly among those who had previously been infected with influenza [59] or pneumonia [85]. In addition, individuals with a history of hospitalization for influenza [60,61], mental health issues [42], and those receiving regular medication [57] were also more likely to receive SIV. The association between general health status and SIV uptake was inconsistent: three studies suggested that individuals with better health status were more likely to receive SIV [65,71,85], while eight studies reported the opposite, suggesting that individuals with poorer health status were more likely to receive SIV [27,42,45,46,54,58,62,84].

#### 3.3.3. Lifestyle and History of Vaccination

Healthy lifestyles were widely recognized to promote SIV uptake, such as regular medical checkups [42,46,47,85], exercise [85], wearing seat belts [46], and wearing masks [48,64]. For other lifestyle factors, such as smoking and alcohol drinking, the findings varied: four studies reported that smoking was associated with lower SIV uptake [42,46,67,85], whereas one study found it to be associated with higher SIV uptake [64]. Regarding alcohol consumption, one study reported an association with lower SIV uptake [67], while two studies found it to be associated with higher SIV uptake [27,46]. Both the history of receiving SIV [26,59,71,81] and the history of receiving pneumococcal vaccination [27,42,57,85] served as facilitators for SIV uptake.

#### 3.3.4. Knowledge and Attitudes Toward SIV

Higher levels of knowledge about SIV were identified as facilitators of SIV uptake [51,52,57,58,60,61,69,70,71,81,85]. Perceptions related to seasonal influenza, including perceived susceptibility to seasonal influenza [27,40,51,55,57,58,60,61,65,69,70,71,83,85] and perceived severity of consequences following seasonal influenza infection [27,53,69,71], were facilitators of SIV uptake. Significant barriers to receiving SIV included concerns about vaccine safety [27,31,40,43,51,53,55,57,58,61,65,69,71,83,85], vaccine affordability [22,27,58,60,69,71,83,85], perceived inconvenience of vaccination [22,27,41,68,69], fear of needles [54], and perceived low priority [52]. Perceived greater SIV effectiveness [27,31,43,51,53,55,57,58,65,69,70,85] and higher self-efficacy [27,65,69] were associated with increased uptake. Five studies found that having more attitudes favoring SIV was associated with higher SIV uptake [44,57,60,61,81]. In addition, perceived SIV uptake as a social responsibility [31,40,51,53,57,69,70] and greater trust in health professionals [31,41,61,69,71] were facilitators of SIV uptake. Receiving cues to action was identified as a significant facilitator of SIV uptake in 11 studies [27,48,51,54,61,65,69,71,83,84,85], such as receiving advice from health professionals [61], getting recommendations from family or peers [69], and participating in community health programs [51].

#### 3.3.5. COVID-19-Related Factors

History of COVID-19 infection [85] and history of COVID-19 vaccination [52,57,59,67,71,85] were associated with higher SIV uptake. Sulis et al. found that less concern about COVID-19 and negative experiences of COVID-19 were facilitators of SIV uptake [26]. Liang et al. found that older adults who perceived a high risk of co-infection with influenza and COVID-19 were more likely to receive a SIV, whereas those who had concerns about potential interactions between SIV and COVID-19 vaccination were less likely to receive SIV [27].

#### 3.3.6. Social Influence

Individuals aged 50 years and older who thoughtfully considered the veracity of vaccine-specific information [44,57] were more likely to receive SIV. In addition, people who had positive experiences with healthcare services were also more likely to receive a SIV [66,69]. Findings related to vaccination intention were similar to those for SIV uptake and are presented in Supplement S3.

#### 3.3.7. Subgroup Analysis

##### Determinants of SIV Uptake Among People Aged 50–64 Years and 65 Years or Above

Only one study reported specific determinants of SIV uptake among adults aged 50–64 years, while 12 studies focused on adults aged 65 and older. Similar determinants were applicable to both subgroups (Supplement S4, Table S6).

##### Determinants of SIV Uptake in Regions Providing Fully Subsidized and Self-Financed SIV

There were 15 studies that reported determinants of SIV uptake in regions providing fully subsidized SIV, and 11 reported determinants in places providing self-financed SIV. Concerns about affordability of the vaccine [22,58,60,69,83] and perceived inconvenience of receiving the vaccine [22,69,83] were common barriers to SIV uptake in regions providing self-financed/partially subsidized SIV, but not in those with fully subsidized SIV (Supplement S4, Table S7).

##### Determinants of SIV Uptake During and After the COVID-19 Pandemic

Twenty-two studies examined determinants of SIV uptake during the COVID-19 pandemic, 12 studies examined determinants after the pandemic, and one study covered both periods. Compared with during the pandemic, perceived susceptibility was more frequently reported as a facilitator after the pandemic [27,57,60,65,69,83,85], whereas concerns about vaccine safety were also more frequently identified as barriers [27,57,65,69,83,85] (Supplement S4, Table S8).

### 3.4. Qualitative Findings

Qualitative studies suggested that reasons for receiving SIV were mainly related to knowledge, attitudes, and social influence. Similar to the quantitative findings, participants reported that a better and more accurate understanding of SIV [63,70,71], perceiving themselves as highly susceptible to influenza and as a priority group for vaccination [51], and thinking that influenza is a serious illness [53,71] encouraged them to get vaccinated. Beliefs that SIV could protect their own health [43,53,55,70] and others’ health [51,53,63,70], the high availability of SIV in community and workplace settings [63,73] and trust in health workers [31,41,63] were also commonly described as reasons for vaccination. Other reasons included receiving recommendations from healthcare providers, family members, or friends [51,73], not experiencing any severe influenza events after receiving SIV [31], and mainstream media coverage of vaccines [31].

However, worrying about vaccine side effects [31,43,51,53,55,71], thinking that there was no need to receive SIV because they considered themselves to be in good health [55,70], believing that SIV was inconvenient [41], feeling unwell after vaccination [63], and perceiving SIV costs as high compared to income or as not covered by insurance [51,71,73] were cited as reasons for not receiving SIV (Supplement S5).

### 3.5. Meta-Analysis

**Figure 2:**
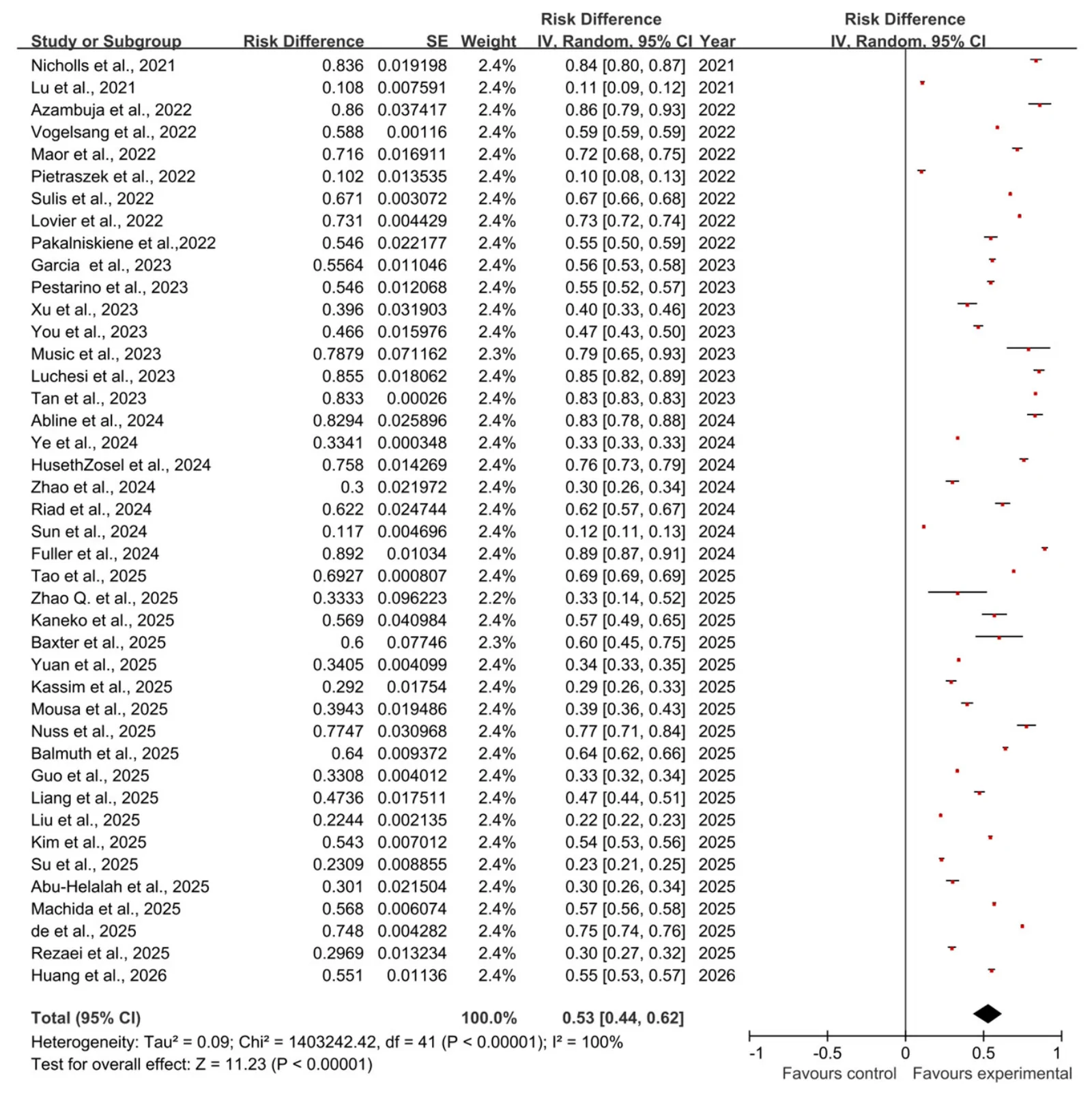
Forest plot of the pooled prevalence for SIV uptake. Colored squares represent the risk-difference estimates of individual studies, with their size indicating the proportion of weight assigned to the study, and the horizontal lines indicating the corresponding 95% CIs. The black diamond represents the pooled risk-difference estimate, with its center and width indicating the pooled estimate and the corresponding 95% CI. CI—confidence interval; IV—inverse variance; SE—standard error [22,26,27,31,40,41,42,43,44,45,46,47,48,49,50,51,52,53,54,55,57,58,59,60,61,62,63,64,65,66,67,68,69,70,71,72,73,81,82,83,84,85].

**Figure 3:**
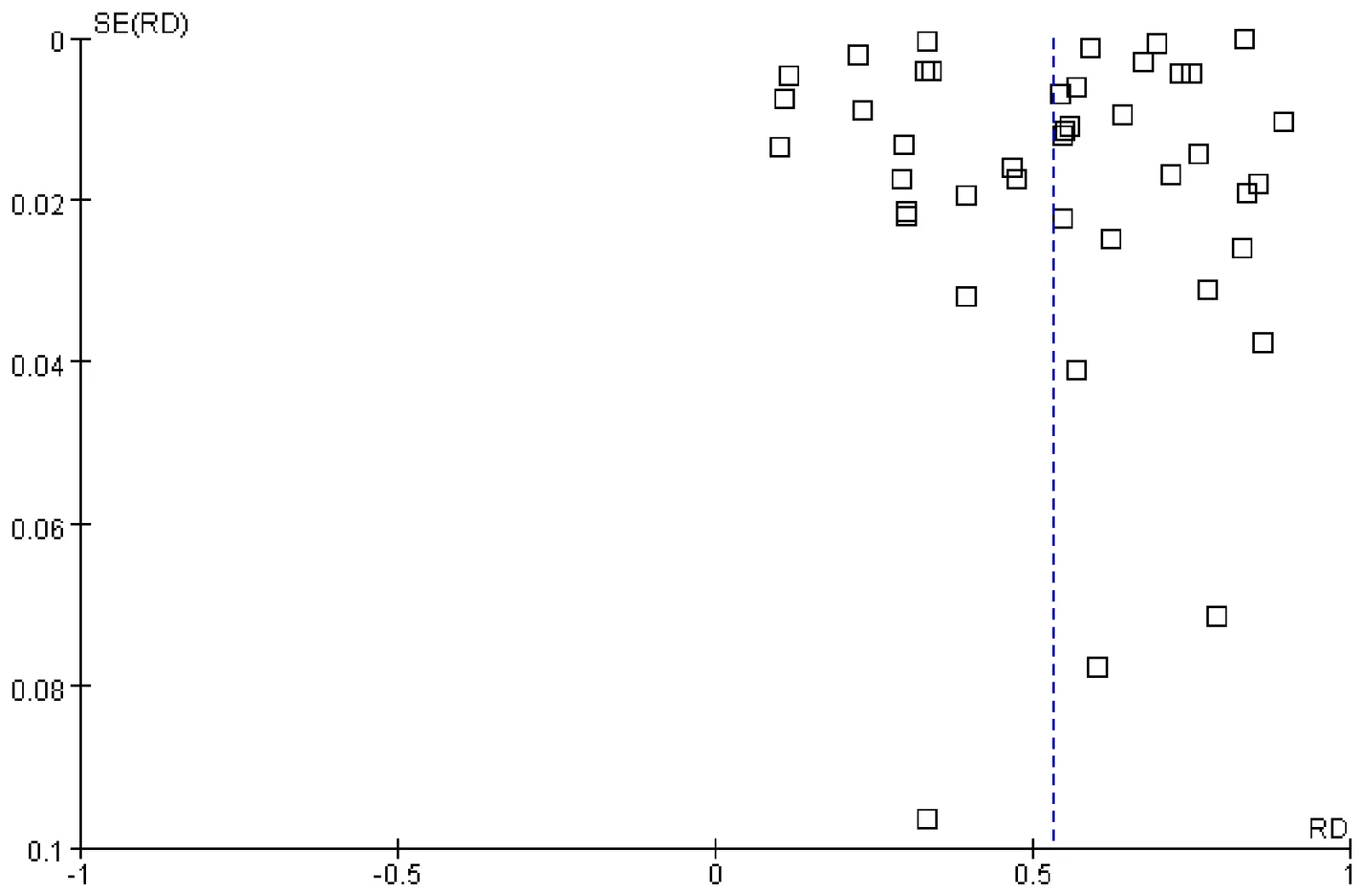
Funnel plot of publication bias among the included studies on SIV uptake. Each square represents an individual study. The blue dashed vertical line indicates the pooled risk-difference estimate. RD—risk difference; SE—standard error.

Among the 43 studies reporting SIV uptake, one study assessed repeated SIV uptake and was therefore not included in the meta-analysis. A total of 42 studies were included in the meta-analysis of SIV uptake, involving 4,548,545 adults aged 50 years and older. The pooled prevalence of SIV uptake was 53% (95% CI: 44–62%), with no publication bias observed across studies, and significant heterogeneity was detected (Table 5, Figure 2 and Figure 3). Subgroup analysis showed that the pooled prevalence of SIV uptake among adults aged 50–64 years (pooled prevalence = 54%, 95% CI: 52–57%; *I*^2^ = 74%, *p* < 0.01) was lower than that among those aged 65 years and older (pooled prevalence = 69%, 95% CI: 64–73%; *I*^2^ = 100%, *p* < 0.001). Significant regional differences were also observed: the highest prevalence was reported in the Americas Region (pooled prevalence = 73%, 95% CI: 69–77%; *I*^2^ = 100%, *p* < 0.001), followed by the European Region (pooled prevalence = 63%, 95% CI: 52–74%; *I*^2^ = 100%, *p* < 0.001) and the Eastern Mediterranean Region (pooled prevalence = 43%, 95% CI: 22–63%; *I*^2^ = 99%, *p* < 0.001), and the lowest in the Western Pacific Region (pooled prevalence = 36%, 95% CI: 32–41%; *I*^2^ = 100%, *p* < 0.001). Under the policy of fully subsidized SIV coverage (pooled prevalence = 71%, 95% CI: 67–75%; *I*^2^ = 100%, *p* < 0.001), SIV uptake prevalence was more than twice as high as in regions with self-financed/partially subsidized policies (pooled prevalence = 33%, 95% CI: 25–42%; *I*^2^ = 100%, *p* < 0.001). SIV uptake prevalence during the COVID-19 pandemic (pooled prevalence = 59%, 95% CI: 52–66%; *I*^2^ = 100%, *p* < 0.001) was higher than that after the COVID-19 pandemic (pooled prevalence = 42%, 95% CI: 37–47%; *I*^2^ = 100%, *p* < 0.001) (Supplement S6). A total of 13 studies that reported SIV intention were included in the meta-analysis of SIV intention, and the results showed patterns similar to those observed for uptake (Supplement S7).

## 4. Discussion

### 4.1. Summary of the Study

This is one of the first systematic reviews to summarize the determinants of SIV uptake among people aged 50 and older during and after the COVID-19 pandemic. The findings revealed the influences of COVID-19-related factors on SIV uptake in this age group. The subgroup analyses identified differences in determinants of SIV uptake between people aged 50–64 years and those aged 65 years or above, between places providing self-financed and fully subsidized SIV, and between studies with data collected during and after the COVID-19 pandemic. These findings could inform service planning and interventions promoting SIV uptake.

### 4.2. Determinants of SIV Uptake: COVID-19-Related Factors

Our findings suggested that some COVID-19-related factors might influence SIV uptake among people aged 50 years or above. Perceived risk of co-infection with SARS-CoV-2 and seasonal influenza was identified as a facilitator of SIV uptake, which was consistent with the findings of another systematic review on determinants of SIV uptake among healthcare workers [86]. The consequences of co-infection were more severe than infection with either SARS-CoV-2 or seasonal influenza alone, which might increase perceived threat of co-infection and hence motivation to receive SIV [87]. Health communication messages may highlight the risk of such co-infection to promote SIV uptake. Moreover, concerns about potential interactions between SIV and COVID-19 vaccination may heighten worries about additive side effects, immune interference, or reduced vaccine effectiveness, which were therefore identified as barriers to SIV uptake. However, recent evidence indicates that the co-administration of the COVID-19 vaccine and SIV is both safe and effective [88]. Similar misconceptions were a barrier to receiving SIV among pregnant women [89], general adults [90], and even healthcare workers [91]. Therefore, it is important to disseminate accurate information regarding the co-administration of the COVID-19 vaccine and SIV in future health promotion. Furthermore, prior history of COVID-19 vaccination was also associated with higher SIV uptake in this age group. People with a prior history of COVID-19 vaccination may have better confidence in vaccines and higher self-efficacy for vaccination [92,93].

### 4.3. Determinants of SIV Uptake: Modifiable Factors

This study also identified some modifiable determinants from both quantitative and qualitative evidence, which were similar to those found by systematic reviews targeting older adults before the COVID-19 outbreak [23,32,33]. Both evidence types identified knowledge of SIV, perceived susceptibility to and severity of influenza, perceived vaccine effectiveness and safety, recommendations from trusted individuals, affordability, and accessibility as important determinants. The two types of evidence provided complementary insights: quantitative studies identified recurring associations between these determinants and SIV uptake or intention across multiple settings, whereas the qualitative findings further illustrated how these determinants influenced decision-making. For example, social responsibility was identified as a facilitator in quantitative studies, and qualitative findings further showed that this responsibility was often expressed as a desire to protect family members and peers. Similarly, concerns about affordability and accessibility were commonly identified as barriers in quantitative studies, and qualitative evidence indicated that these barriers were experienced in more concrete ways, such as the perceived cost of vaccination relative to income or insurance coverage, and the availability of vaccination in community or workplace settings. Qualitative findings also highlighted the influence of trust in healthcare workers, previous post-vaccination experiences, and media coverage, which helped explain why individuals with similar demographic or health profiles might make different vaccination decisions. Thus, the qualitative evidence complemented and contextualized the associations identified in the quantitative studies. These determinants should be considered when promoting SIV uptake in the post-pandemic era.

### 4.4. Subgroup Analysis

In the subgroup analysis among people aged 50–64 years and those aged 65 years or above, other studies suggested that middle-aged adults may be more likely to be influenced by workplace-related factors than older adults, such as risk of influenza infection at the workplace [94], impacts of influenza on work performance and income [95], and inconvenience of receiving a SIV due to busy schedules [94]. However, we were not able to draw a conclusion about whether determinants were different between these two age groups as there was a dearth of quantitative studies investigating facilitators or barriers that were specific to people aged 50–64 years. More studies are needed to explore determinants of SIV uptake among people aged 50–64 years, and to investigate whether such determinants are similar to those among people aged 65 years or above to inform health promotion tailored to the needs of middle-aged adults. Subgroup analysis in places without fully subsidized SIV revealed that cost and accessibility were barriers to receiving SIV among both people aged 50–64 years and those aged 65 years or above. Therefore, policy efforts to improve SIV uptake should reduce financial and logistical barriers through subsidized vaccination, simplified appointment procedures, and workplace vaccination. The emergence of the COVID-19 pandemic may have heightened people’s perceived susceptibility to respiratory infections, but the subsequent misinformation about COVID-19 vaccines has, in turn, increased concerns about vaccine safety [96]. Therefore, health authorities should raise awareness about susceptibility to respiratory infections and debunk misinformation about vaccines.

### 4.5. Meta-Analysis

The pooled prevalence of SIV uptake was higher than the reported global estimates among children aged 6–59 months [97] and the general population [98]. Although people aged 50 and above are a priority group for SIV, SIV coverage remains below the WHO’s target of 75% coverage [19]. Subgroup analyses showed that the pooled prevalence of SIV uptake was lower among adults aged 50–64 years than among adults aged 65 years and above. There are some possible reasons to explain the difference. Adults aged 65 years and above usually perceive greater susceptibility to and severity of seasonal influenza [99], while adults aged 50–64 years may perceive themselves as less vulnerable [99], be less aware of their eligibility to receive fully subsidized SIV [52,100] and face workplace-related barriers [94,95]. In addition, both the SIV uptake rate and level of intention to receive a SIV were lower in places without fully subsidized SIV compared with those with fully subsidized vaccines. Providing fully subsidized SIV can not only reduce practical barriers, such as cost and limited accessibility [51,101], but also increase awareness of eligibility and public trust [75,80]. Our subgroup analysis also indicated regional differences in SIV coverage. Higher SIV uptake was observed in the Americas and European Regions. These regions may have more mature immunization platforms, clearer vaccination recommendations, stronger public financing and higher vaccine availability [16,102]. Lower coverage was observed in the Western Pacific Region, which might be attributed to the limited or unstable vaccine supply, weaker healthcare systems, lower trust in healthcare services, and lack of targeted communications [103,104,105]. There is a gap in SIV uptake coverage between studies with data collected during and after the COVID-19 pandemic, which is similar to the findings of another global study [106]. The higher SIV uptake during the COVID-19 pandemic may be attributed to heightened risk perception [26], increased awareness of vaccination benefits [28], and strengthened public health promotion [107]. However, after the pandemic, vaccination patterns returned to pre-pandemic conditions, and vaccine fatigue and persistent safety concerns caused by the pandemic may have further reduced SIV uptake [30,31]. There was high heterogeneity in the pooled prevalence of SIV uptake among adults aged 50 years and above. Although subgroup analyses were conducted according to age group, geographic regions, and vaccination policy context, substantial heterogeneity remained. This residual heterogeneity may be attributable to some other factors, such as different sampling methods (community-based or hospital-registry-based), outcome measurement (self-reported or medical-record-based), outcome timeframe (in the last flu season or any season during the lifetime), and influenza seasons (20/21 season to 24/25 season). Therefore, the pooled prevalence should be interpreted as a broad summary rather than a precise universal estimate.

### 4.6. Limitations

This study still has several limitations. First, the findings should be interpreted with caution due to high heterogeneity in the pooled prevalence of SIV uptake. Second, the study outcomes in this review were self-reported SIV uptake or intention, which were not officially verified in some studies, and may be subject to recall bias and social desirability bias. Third, since all of the included studies were observational studies, it is not certain whether there is a causal relationship between associated factors and SIV uptake. Fourth, there are limited quantitative studies specifically targeting adults aged 50–64 years, and most findings for this age group are derived from age-stratified results. This limits the applicability of the findings from the age-subgroup analysis. Fifth, the restriction to studies involving adults aged ≥50 years may have contributed to the limited number of included studies and potential underrepresentation of certain regions. Given that there are still 26 countries worldwide with an average life expectancy at birth below 65 years [108], it is less likely that these countries would report determinants of SIV among people aged 50 years or older. Finally, this review included only studies published in English because the review team lacked the multilingual expertise needed to verify the accuracy of translated full texts. Although the available tools can easily translate non-English content, they may misinterpret complex academic terminology [109]. Previous studies suggested that English-language studies were of higher methodological quality than those published in other languages [110]. Restricting systematic reviews to English-language publications appears to have little impact on the effect estimates and conclusions of systematic reviews, especially for standard health-related topics like ours [111,112].

## 5. Conclusions

Policymakers and health authorities should adapt SIV promotion to the post-pandemic context by addressing concerns about SARS-CoV-2 and influenza co-infection and the safety of co-administration of COVID-19 vaccines and SIV. Evidence-based communication through trusted healthcare professionals should be combined with established strategies targeting modifiable determinants, including knowledge, risk perceptions, vaccine confidence, provider recommendations, and access to vaccination. As SIV programs expand to include adults aged 50–64 years, further research is needed to identify facilitators and barriers specific to this group and inform tailored communication and service-delivery strategies.

## Figures and Tables

**Table 1:** Search strategies.

Steps	Search Terms
1	influenza
2	vaccin* OR immun*OR inoculation OR shot*
3	“middle aged” OR “middle-aged” OR “older adult*” OR “50 years” OR “50 years and older” OR “aged 50” OR old people OR old adults OR elder* OR older people OR older adults OR senior* OR seniors OR geriatric
4	willing* OR accept* OR refus* OR hesitan* OR inten* OR reject* OR declin* OR uptake* OR factor* OR determinant* OR reason* OR facilitator* OR barrier* OR attitude* OR perception* OR view* OR predict* OR enabl* OR knowledge OR perspective*
5	#1 AND #2 AND #3 AND #4

**Table 2:** Inclusion and exclusion criteria.

Parameter	Inclusion Criteria	Exclusion Criteria
Population (P)	➢ Studies including adults aged 50 years or above ➢ Studies reporting results among subgroups of adults aged 50 years or above	➢ Studies focusing on the specific group (e.g., healthcare workers, patients with a certain disease) ➢ Studies not focusing on the population level (e.g., laboratory simulations, clinician-only samples)
Exposure (E)	➢ Studies reporting qualitative determinants and/or quantitative reasons related to SIV uptake or intention	➢ None
Outcomes (O)	➢ Studies reporting quantitative or qualitative outcomes related to SIV uptake and/or intention	➢ Studies only focusing on vaccine efficacy, effectiveness, immunogenicity, or safety
Study setting	➢ No limit	➢ None
Study design	➢ Observational quantitative studies (e.g., cross-sectional studies, cohort studies and case–control studies) ➢ Observational qualitative studies ➢ Mixed-methods studies with relevant quantitative or qualitative data	➢ Intervention studies (e.g., randomized controlled trials, quasi-experimental studies and single-arm studies) ➢ Reviews, narratives, commentaries, or editorials ➢ Dissertations, preprints, government reports, newspaper articles, textbooks, book chapters, and protocols ➢ Laboratory studies, model and framework studies, and validation studies
Language	➢ English language	➢ None
Publication period	➢ Between 1 December 2019 and 1 January 2026	➢ None
Availability of full text	➢ Full text is available and accessible	➢ None

**Table 3:** Characteristics of included studies.

Author, Year	Country	Policy Context of SIV	Design	Sample Size	Age Group	Outcome
Lu et al., 2021 [22]	China	Self-financed	Cross-sectional study	1672	50–69	SIV uptake: 10.80%; SIV intention: 70.50%
Nicholls et al., 2021 [40]	United Kingdom	Fully subsidized	Cross-sectional study	372	65+	SIV uptake 83.60%
Waite et al., 2021 [74]	Canada	Not reported	Mixed-methods study	4501 (50–64: 1001; 65+: 3500)	50+ (50–64 & 65+)	SIV intention 77.31% (50–64: 67.00%; 65+: 80.20%)
Azambuja et al., 2022 [41]	Brazil	Fully subsidized	Cross-sectional study with qualitative data	86	60+	SIV uptake 86.00%
Che et al., 2022 [75]	China	Fully subsidized	Cross-sectional study	11,663	70+	SIV intention 85.98%
Lovier et al., 2022 [42]	United States	Fully subsidized	Cross-sectional study	10,025	65+	SIV uptake 73.10%
Maor et al., 2022 [43]	Israel	Fully subsidized	Cross-sectional study with qualitative data	711	65+ subgroup	SIV uptake 71.60%
Pakalniskiene et al., 2022 [44]	Lithuania	Not reported	Cross-sectional study	504	50+	SIV uptake 54.60%
Pietraszek et al., 2022 [45]	Poland	Partially subsidized	Cross-sectional study	500	60+	SIV uptake 10.20%
Sulis et al., 2022 [26]	Canada	Fully subsidized for 65+	Cross-sectional study	23,385 (50–64: 8160; 65+: 15,225)	50+ (50–64, 65+)	SIV uptake 67.10% (50–64: 52.86%; 65+: 74.73%)
Vogelsang et al., 2022 [46]	United States	Not reported	Cross-sectional study	180,064	60+	SIV uptake 58.80%
García-Hernández et al., 2023 [47]	Mexico	Fully subsidized	Cross-sectional study	2023	65+	SIV uptake 55.64%
Liang et al., 2023 [76]	China	Fully subsidized	Cross-sectional study	440	65+	SIV intention 55.70%
Luchesi et al., 2023 [48]	Brazil	Fully subsidized	Cross-sectional study	380	60+	SIV uptake 85.50%
Music et al., 2023 [31]	Canada	Fully subsidized	Mixed-methods study	33	65+	SIV uptake 78.79%
Pestarino et al., 2023 [49]	Italy	Fully subsidized	Cross-sectional study	1702	60+	SIV uptake 54.60%
Tan et al., 2023 [50]	England	Fully subsidized	Cross-sectional study	2,054,463	65+	SIV uptake 83.30%
Xu et al., 2023 [51]	Singapore	Partially subsidized	Mixed-methods study	235	65+	SIV uptake 39.60%
You et al., 2023 [52]	China	Fully subsidized	Cross-sectional study	975	60+	SIV uptake 46.60%
Abline et al., 2024 [53]	France	Fully subsidized	Cross-sectional study with qualitative data	211	65+	SIV uptake 82.94%
Fuller et al., 2024 [54]	United States	Fully subsidized	Cross-sectional study	901	65+	SIV uptake 89.20%
Huseth-Zosel et al., 2024 [55]	United States	Fully subsidized	Cross-sectional study with qualitative data	901	65+	SIV uptake 75.80%
Niu et al., 2024 [56]	China	Self-financed	Cross-sectional study	29,200	60+	Repeated SIV uptake: Once: 20.55%; Twice: 13.70%; 3 times: 10.27%
Riad et al., 2024 [57]	Czech Republic	Fully subsidized for 65+	Cross-sectional study	384	55+	SIV uptake 72.80%
Sun et al., 2024 [58]	China	Self-financed	Cross-sectional study	4684	60+	SIV uptake 11.70%
Ye et al., 2024 [59]	China	Fully subsidized for 65+	Cross-sectional study	1,838,794	60+	SIV uptake 33.41%
Zhang et al., 2024 [77]	China	Self-financed	Cross-sectional study	3138	60–75	SIV intention 61.30%
Zhao et al., 2024 [60]	China	Self-financed	Cross-sectional study	435	60+	SIV uptake 30.00%
Abu-Helalah et al., 2025 [61]	Jordan	Self-financed	Cross-sectional study	455	65+	SIV uptake 30.10%
Balmuth et al., 2025 [62]	United States	Not reported	Cross-sectional study	2623 (50–64: 1375; 65+: 1248)	50+ (50–64, 65+)	SIV uptake 64.00% (50–64: 52.80%; 65+: 75.35%)
Baxter et al., 2025 [63]	England and Scotland	Fully subsidized	Qualitative study	40	50–64	SIV uptake 60.00%
de et al., 2025 [64]	Spain	Fully subsidized	Cross-sectional study	10,279	65+	SIV uptake 74.80%
Guo et al., 2025 [65]	China	Depending on the city, either Fully subsidized or Self-financed.	Cross-sectional study	13,754	60+	SIV uptake 33.08%
Kaneko et al., 2025 [66]	Japan	Not reported	Cross-sectional study	146	65+ subgroup	SIV uptake 56.90%
Kassim et al., 2025 [81]	Malaysia	Self-financed	Cross-sectional study	672	60+	SIV uptake: 29.20%; SIV intention: 62.30%
Kim et al., 2025 [67]	Korea	Self-financed	Cross-sectional study	5047	50–64 subgroup	SIV uptake 54.30%
Liang et al., 2025 [27]	China	Fully subsidized	Cross-sectional study	813 (2021–2022: 440, 2023–2024: 373)	65+	SIV uptake 47.36% (2021–2022: 45.00%; 2023–2024: 50.10%)
Liu et al., 2025 [82]	China	Self-financed	Cross-sectional study	38,184	60+	SIV uptake 22.44%; SIV intention 73.84%
Machida et al., 2025 [68]	Japan	Not reported	Cross-sectional study	6651	65+	SIV uptake 56.80%
Mousa et al., 2025 [69]	Jordan	Self-financed	Cross-sectional study	629	Older adults subgroup (no specific age group reported)	SIV uptake 39.43%
Nuss et al., 2025 [70]	United States	Fully subsidized	Cross-sectional study with qualitative data	182 (50–64: 101; 65+: 81)	50+ (50–64, 65+) subgroup	SIV uptake 77.47%; (50–64: 69.30%; 65+: 87.70%)
Rezaei et al., 2025 [71]	Iran	Fully subsidized for 65+	Cross-sectional study with qualitative data	1192	60+	SIV uptake 29.69%
Su et al., 2025 [83]	China	Three representative cities: Fully subsidized, Partially subsidized, Self-financed	Cross-sectional study	2265	60+	SIV uptake 23.09% (Fully subsidized: 53.29%; Partially subsidized: 20.85%; Self-financed: 13.60%); SIV intention 46.05% (Fully subsidized: 68.78%; Partially subsidized: 47.71%; Self-financed: 37.15%)
Tao et al., 2025 [72]	United States	Not reported	Cross-sectional study	326,869	65+	SIV uptake 69.27%
Wang et al., 2025 [78]	China	Self-financed	Cross-sectional study	1300	60+	SIV intention 14.80%
Yang et al., 2025 [79]	China	Self-financed	Cross-sectional study	538	60+	SIV intention 80.86%
Yuan et al., 2025 [84]	China	include Fully subsidized and Self-financed, cannot classify	Cross-sectional study	13,363	60+	SIV uptake: 34.05%; SIV intention: 32.20%
Zhao Y. et al., 2025 [73]	China	Fully subsidized	Cross-sectional study	7103	60+	SIV intention 73.15%
Zhao Q. et al., 2025 [80]	China	Self-financed	Qualitative study	24	60+	SIV uptake 33.33%
Huang et al., 2026 [85]	China	Fully subsidized	Cross-sectional study	1917	60+	SIV uptake 55.1%; SIV intention: 77.4%

SIV—seasonal influenza vaccination.

**Table 4:** Determinants of SIV uptake in quantitative studies and quantitative data in mixed-methods studies (n = 35).

		1	2	3	4	5	6	7	8	9	10	11	12	13	14	15	16	17	18	19	20	21	22	23	24	25	26	27	28	29	30	31	32	33	34	35
Socio-demographics	Male	●						●											●									●	●							
	Older age						●	●		●		●			●	●			●	●		●						●	●							●
	Local residents	●																	●																	●
	Living in urban area																		●		●	●	●						●							
	Living at nursing home/sanatorium																												●							
	Higher income			●		●	●	●					●		●			●		●	●	●		●					●			●		●	●	●
	Higher education						●									●		●			●	●	●	●					●			●		●		
	Married							●																●			●					●				●
	Living with family							●					●	●				●					●					●								
	Employment							●													●							●				●				●
	Health insurance coverage								●								●		●		●			●												●
Health conditions	General health status			●		●		●								●		●				●		●				●				●			●	●
	Having chronic disease (e.g., cardiovascular disease, hyperlipidemia)	●					●		●			●	●				●	●						●			●	●				●				
	History of vaccine-preventable diseases (e.g., influenza, pneumonia)	●																	●	●	●															●
	Having mental health issues (e.g., anxiety, depression)			●																																
	History of hospitalization due to influenza																			●	●															
	Receiving medication regularly																●																			
Lifestyles	Regular medical checkup			●				●	●																											●
	Smoking			●				●															●				●									●
	Alcohol drinking							●																			●	●								
	Exercise																																			●
	Wearing seat belt							●																												
	Wearing masks									●													●													
History of vaccination	Pneumonia vaccination			●													●											●								●
	SIV						●												●							●						●				
Knowledge and attitudes	General positive attitudes towards vaccination				●												●			●	●					●										
	Perceived disease susceptibility		●											●			●	●		●	●			●				●			●		●			●
	Perceived disease severity																											●			●		●			
	Perceived vaccine effectiveness										●			●			●	●						●				●			●					●
	Concern of vaccine safety		●								●			●			●	●			●			●				●			●		●			●
	Concerns of vaccine affordability	●																●		●								●			●		●			●
	Perceived inconvenience of vaccination	●																										●		●	●		●			
	Fear of needles															●																	●			
	Perceived low priority														●																					
	Perceived self-efficacy																							●				●			●					
	Higher level of vaccine knowledge													●	●		●	●		●	●					●					●		●			●
	Perceived vaccination as a social responsibility		●								●			●			●														●					
	Trust to health professionals										●										●										●	●				
	Received health professional/family/community recommendation									●				●		●					●			●				●			●	●	●		●	●
Factors that are relevant to COVID-20	History of COVID-19 infection																																			●
	History of COVID-19 vaccination														●		●		●								●					●				●
	Concerns about COVID-19						●																													
	Negative experience of COVID-19						●																													
	Perceived high risk of co-infection of SIV and COVID-19																											●								
	Concerns about the potential interactions of SIV and COVID-19 vaccination																											●								
Social influence	Social media promotion																							●												
	Thoughtful consideration of the veracity of vaccine-specific information				●												●																			
	Positive experience of healthcare service																								●						●					

● Facilitator, ● Barriers. 1: Lu et al. [22]; 2: Nicholls et al., 2021 [40]; 3: Lovier et al., 2022 [42]; 4: Pakalniskiene et al., 2022 [44]; 5: Pietraszek et al., 2022 [45]; 6: Sulis et al., 2022 [26]; 7: Vogelsang et al., 2022 [46]; 8: García-Hernández et al., 2023 [47]; 9: Luchesi et al., 2023 [76]; 10: Music et al., 2023 [31]; 11: Pestarino et al., 2023 [49]; 12: Tan et al., 2023 [50]; 13: Xu et al., 2023 [51]; 14: You et al., 2023 [52]; 15: Fuller et al., 2024 [54]; 16: Riad et al., 2024 [57]; 17: Sun et al., 2024 [58]; 18: Ye et al., 2024 [59]; 19: Zhao et al., 2024 [60]; 20: Abu-Helalah et al., 2025 [61]; 21: Balmuth et al., 2025 [62]; 22: de et al., 2025 [64]; 23: Guo et al., 2025 [65]; 24: Kaneko et al., 2025 [66]; 25: Kassim et al., 2025 [81]; 26: Kim et al., 2025 [67]; 27: Liang et al., 2025 [27]; 28: Liu et al., 2025 [82]; 29: Machida et al., 2025 [68]; 30: Mousa et al., 2025 [69]; 31: Rezaei et al., 2025 [71]; 32: Su et al., 2025 [83]; 33: Tao et al., 2025 [72]; 34: Yuan et al., 2025 [84]; 35: Huang et al., 2026 [85].

**Table 5:** Meta-analysis results for pooled prevalence of SIV uptake and intention.

Outcome/Subgroup	Studies Number	Pooled Prevalence	Heterogeneity, *I*^2^	Publication Bias, Beta (SE)	Subgroup Difference
**SIV uptake**	**42**	**0.53 [0.44, 0.62]**	100% ***	**−0.06 (0.23)**	
**Age group**					**<0.001**
50–64 years	5	0.54 [0.52, 0.57]	74% **	0.08 (0.04) *	
65+ years	19	0.69 [0.64, 0.73]	100% ***	0.70 (0.26)	
**Region**					**<0.001**
Americas Region	12	0.73 [0.69, 0.77]	100% ***	0.77 (0.19) *	
Eastern Mediterranean Region	4	0.43 [0.22, 0.63]	99% ***	−0.42 (3.14)	
European Region	9	0.63 [0.52, 0.74]	100% ***	0.81 (0.68)	
Western Pacific Region	17	0.36 [0.32, 0.41]	100% ***	−0.66 (0.24)	
**Policy context**					**<0.001**
Fully subsidized	22	0.71 [0.67, 0.75]	100% ***	0.74 (0.2)	
Self-financed/partially subsidized	15	0.33 [0.25, 0.42]	100% ***	−0.91 (0.36)	
**Vaccination season**					**<0.001**
During the COVID-19 pandemic	28	0.59 [0.52, 0.66]	100% ***	0.07 (0.33)	
After the COVID-19 pandemic	15	0.42 [0.37, 0.47]	100% ***	−0.39 (0.21)	
**SIV intention**	**13**	**0.65 [0.58, 0.73]**	100% ***	**0.75 (0.56)**	
**Policy context**					**<0.001**
Fully subsidized	6	0.74 [0.68, 0.80]	99% ***	1.80 (0.33) *	
Self-financed/partially subsidized	8	0.59 [0.44, 0.74]	100% ***	0.45 (0.91)	
**Vaccination season**					**<0.001**
During the COVID-19 pandemic	5	0.73 [0.66, 0.80]	100% ***	1.66 (0.34)	
After the COVID-19 pandemic	8	0.56 [0.39, 0.73]	100% ***	−0.09 (0.88)	

* *p* < 0.05, ** *p* < 0.01, and *** *p* < 0.001. SE—standard error; SIV—seasonal influenza vaccination.

## Data Availability

Data supporting this systematic review are available in the reference section. In addition, the analyzed data used in this systematic review are available from the author upon reasonable request.

## Supplementary Materials

The following supporting information can be downloaded at: https://www.mdpi.com/article/10.3390/vaccines14080705/s1, Supplement S1. Search strategies: Table S1. Search strategies across nine databases. Supplement S2. Quality assessments of included studies: Table S2. Quality assessments included quantitative non-randomized studies (assessed by MMAT version 2018). Table S3. Quality assessments included qualitative studies (assessed by MMAT version 2018). Table S4. Quality assessments included mixed-methods studies (assessed by MMAT version 2018). Supplement S3. Determinants of SIV intention in quantitative and qualitative studies: Table S5. Determinants of SIV intention in quantitative and qualitative studies determinants of SIV intention in all studies (n = 13). Supplement S4. Subgroup analyses of determinants of SIV uptake in quantitative studies: Table S6. Determinants of SIV uptake by age group. Table S7. Determinants of SIV uptake by vaccination policy context. Table S8. Determinants of SIV uptake by vaccination season. Supplement S5. Reasons for SIV uptake in qualitative data: Table S9. Reasons for SIV uptake in qualitative data (n = 10). Supplement S6. Subgroup analysis of meta-analysis on SIV uptake: Figure S1. Forest plot for subgroup analysis of pooled prevalence of SIV uptake: (a) age group; (b) region; (c) policy context; (d) vaccination season. Figure S2. Funnel plot of publication bias for subgroup analyses of SIV uptake (a) age group; (b) region; (c) policy context; (d) vaccination season. Supplement S7. Meta-analysis on SIV intention and its subgroup analysis: Figure S3. Forest plot of the pooled prevalence for SIV intention. Figure S4. Funnel plot of publication bias among the included studies on SIV intention. Figure S5. Forest plot for subgroup analysis of pooled prevalence of SIV intention: (a) policy context; (b) vaccination season. Figure S6. Funnel plot of publication bias for subgroup analyses of SIV intention (a) policy context; (b) vaccination season.
