# Supplementary material for: Determinants of the Seasonal Influenza Vaccination Uptake Among People Aged 50 Years or Above During and After the Pandemic: A Systematic Review
Source: Vaccines (Basel). 2026 Aug 15;14(8):705. doi: 10.3390/vaccines14080705 (PMC13517376; doi:10.3390/vaccines14080705)
Supplement: Supplementary file 1 [file vaccines-14-00705-s001.zip › vaccines-4449996-supplementary.pdf]

List of Supplementary Materials

Supplement S1. Search strategies. 2

Table S1. Search strategies across nine databases. 2

Supplement S2. Quality assessments of included studies. 5

Table S2. Quality assessments of included quantitative non-randomized studies (assessed by MMAT version 2018). 5

Table S3. Quality assessments of included qualitative studies (assessed by MMAT version 2018). 7

Table S4. Quality assessments of included mixed methods studies (assessed by MMAT version 2018). 8

Supplement S3. Determinants of SIV intention in quantitative and qualitative studies. 9

Table S5. Determinants of SIV intention in quantitative and qualitative studies determinants of SIV intention in all studies (n=13). 11

Supplement S4. Subgroup analyses of determinants of SIV uptake in quantitative studies 14

Table S6. Determinants of SIV uptake by age group 14

Table S7. Determinants of SIV uptake by vaccination policy context. 17

Table S8. Determinants of SIV uptake by vaccination season. 22

Supplement S5. Reasons for SIV uptake in qualitative data 28

Table S9. Reasons for SIV uptake in qualitative data (n=10). 28

Supplement S6. Subgroup analysis of meta-analysis on SIV uptake. 31

Figure S1. Forest plot for subgroup analysis of pooled prevalence of SIV uptake: (a) age group; (b) region; (c) policy context; (d) vaccination season 31

Figure S2. Funnel plot of publication bias for subgroup analyses of SIV uptake (a) age group; (b) region; (c) policy context; (d) vaccination season 32

Supplement S7. Meta-analysis on SIV intention and its subgroup analysis. 33

Figure S3. Forest plot of the pooled prevalence for SIV intention. 33

Figure S4. Funnel plot of publication bias among the included studies on SIV intention. 33

Figure S5. Forest plot for subgroup analysis of pooled prevalence of SIV intention: (a) policy context; (b) vaccination season. 34

Figure S6. Funnel plot of publication bias for subgroup analyses of SIV intention (a) policy context; (b) vaccination season. 35

Supplement S1. Search strategies.

Table S1. Search strategies across nine databases.

Steps	Search terms	Results
a. Pubmed		
1	influenza[Title/Abstract]	21,767
2	vaccin*[Title/Abstract] OR immun*[Title/Abstract] OR inoculation[Title/Abstract] OR shot*[Title/Abstract]	581,364
3	"middle aged"[Title/Abstract] OR "middle-aged"[Title/Abstract] OR "older adult*"[Title/Abstract] OR "50 years"[Title/Abstract] OR "50 years and older"[Title/Abstract] OR "aged 50"[Title/Abstract] OR old people[Title/Abstract] OR old adults[Title/Abstract] OR elder*[Title/Abstract] OR older people[Title/Abstract] OR older adults[Title/Abstract] OR senior*[Title/Abstract] OR seniors[Title/Abstract] OR geriatric[Title/Abstract]	186,409
4	willing*[Title/Abstract] OR accept*[Title/Abstract] OR refus*[Title/Abstract] OR hesitan*[Title/Abstract] OR inten*[Title/Abstract] OR reject*[Title/Abstract] OR declin*[Title/Abstract] OR uptake*[Title/Abstract] OR factor*[Title/Abstract] OR determinant*[Title/Abstract] OR reason*[Title/Abstract] OR facilitator*[Title/Abstract] OR barrier*[Title/Abstract] OR attitude*[Title/Abstract] OR perception*[Title/Abstract] OR view*[Title/Abstract] OR predict*[Title/Abstract] OR enabl*[Title/Abstract] OR knowledge[Title/Abstract] OR perspective*[Title/Abstract]	2,188,071
5	#1 AND #2 AND #3 AND #4	745
b. Medline		
1	TS= influenza	21,487
2	TS= vaccin* OR immun* OR inoculation OR shot*	599,092
3	TS="middle aged" OR "middle-aged" OR "older adult*" OR "50 years" OR "50 years and older" OR "aged 50" OR old people OR old adults OR elder* OR older people OR older adults OR senior* OR seniors OR geriatric	984,590
4	TS= willing* OR accept* OR refus* OR hesitan* OR inten* OR reject* OR declin* OR uptake* OR factor* OR determinant* OR reason* OR facilitator* OR barrier* OR attitude* OR perception* OR view* OR predict* OR enabl* OR knowledge OR perspective*	2,255,285
5	#1 AND #2 AND #3 AND #4	2,038
c. Embase		
1	(influenza) .ti,ab,kw	16,007
2	(vaccin* OR immune* OR inoculation OR shot*).ti,ab,kw	521,061
3	("middle aged" OR "middle-aged" OR "older adult*" OR "50 years" OR "50 years and older" OR "aged 50" OR old people OR old adults OR elder* OR older people OR older adults OR senior* OR seniors OR geriatric).ti,ab,kw	511,172
4	(willing* OR accept* OR refus* OR hesitan* OR inten* OR reject* OR declin* OR uptake* OR factor* OR determinant* OR reason* OR facilitator* OR barrier* OR attitude* OR perception* OR view* OR predict* OR enabl* OR knowledge OR perspective*).ti,ab,kw	1,549,662
5	#1 AND #2 AND #3 AND #4	1,510
d. Web of Science		
1	TS= influenza	37,936
2	TS= vaccin* OR immune* OR inoculation OR shot*	948,702
3	TS="middle aged" OR "middle-aged" OR "older adult*" OR "50 years" OR "50 years and older"	374,186

	OR "aged 50" OR old people OR old adults OR elder* OR older people OR older adults OR senior* OR seniors OR geriatric	
4	TS= willing* OR accept* OR refus* OR hesitan* OR inten* OR reject* OR declin* OR uptake* OR factor* OR determinant* OR reason* OR facilitator* OR barrier* OR attitude* OR perception* OR view* OR predict* OR enabl* OR knowledge OR perspective*	7,252,342
5	#1 AND #2 AND #3 AND #4	1,511
e. Global Health		
1	TI, AB.SU= influenza	5,770
2	TI, AB.SU= vaccin* OR immun* OR inoculation OR shot*	88,159
3	TI, AB.SU="middle aged" OR "middle-aged" OR "older adult*" OR "50 years" OR "50 years and older" OR "aged 50" OR old people OR old adults OR elder* OR older people OR older adults OR senior* OR seniors OR geriatric	23,010
4	TI, AB.SU= willing* OR accept* OR refus* OR hesitan* OR inten* OR reject* OR declin* OR uptake* OR factor* OR determinant* OR reason* OR facilitator* OR barrier* OR attitude* OR perception* OR view* OR predict* OR enabl* OR knowledge OR perspective*	235,677
5	#1 AND #2 AND #3 AND #4	310
f. CINAHL		
1	XB= influenza	886
2	XB= vaccin* OR immune* OR inoculation OR shot*	21,445
3	XB="middle aged" OR "middle-aged" OR "older adult*" OR "50 years" OR "50 years and older" OR "aged 50" OR old people OR old adults OR elder* OR older people OR older adults OR senior* OR seniors OR geriatric	34,543
4	XB= willing* OR accept* OR refus* OR hesitan* OR inten* OR reject* OR declin* OR uptake* OR factor* OR determinant* OR reason* OR facilitator* OR barrier* OR attitude* OR perception* OR view* OR predict* OR enabl* OR knowledge OR perspective*	252,846
5	#1 AND #2 AND #3 AND #4	50
g. Cochrane Library		
1	(influenza) .ti,ab,kw	2,668
2	(vaccin* OR immune* OR inoculation OR shot*).ti,ab,kw	58,204
3	(middle NEXT aged OR middle-aged OR older NEXT adult* OR 50 NEXT years OR 50 NEXT years NEXT older OR aged NEXT 50 OR old people OR old adults OR elder* OR older people OR older adults OR senior* OR seniors OR geriatric).ti,ab,kw	145,300
4	(willing* OR accept* OR refus* OR hesitan* OR inten* OR reject* OR declin* OR uptake* OR factor* OR determinant* OR reason* OR facilitator* OR barrier* OR attitude* OR perception* OR view* OR predict* OR enabl* OR knowledge OR perspective*).ti,ab,kw	303,398
5	#1 AND #2 AND #3 AND #4	317
h. APA PsycINFO		
1	(influenza) .ti,ab,kw	336
2	(vaccin* OR immun* OR inoculation OR shot*).ti,ab,kw	6,568
3	("middle aged" OR "middle-aged" OR "older adult*" OR "50 years" OR "50 years and older" OR "aged 50" OR old people OR old adults OR elder* OR older people OR older adults OR senior* OR seniors OR geriatric).ti,ab,kw	28,438
4	(willing* OR accept* OR refus* OR hesitan* OR inten* OR reject* OR declin* OR uptake* OR factor* OR determinant* OR reason* OR facilitator* OR barrier* OR attitude* OR	188,243

	perception* OR view* OR predict* OR enabl* OR knowledge OR perspective*).ti,ab,kw	
5	#1 AND #2 AND #3 AND #4	38
i. APA PsycArticle		
1	(influenza) .ti,ab,kw	20
2	(vaccin* OR immune* OR inoculation OR shot*).ti,ab,kw	330
3	("middle aged" OR "middle-aged" OR "older adult*" OR "50 years" OR "50 years and older" OR "aged 50" OR old people OR old adults OR elder* OR older people OR older adults OR senior* OR seniors OR geriatric).ti,ab,kw	2,121
4	(willing* OR accept* OR refus* OR hesitan* OR inten* OR reject* OR declin* OR uptake* OR factor* OR determinant* OR reason* OR facilitator* OR barrier* OR attitude* OR perception* OR view* OR predict* OR enabl* OR knowledge OR perspective*).ti,ab,kw	23,901
5	#1 AND #2 AND #3 AND #4	3

Supplement S2. Quality assessments of included studies.

Table S2. Quality assessments of included quantitative non-randomized studies (assessed by MMAT version 2018).

MMAT Question-Quantitative nonrandomized studies	1	2	3	4	5	6	7	8	9	10	11	12	13	14	15	16	17	18	19	20	21	22	23	24	25	26	27	28	29	30	31	32	33	34	35	36	37	38	39	40	41	42	43	44	45	46
S1. Are there clear research questions?	Y	Y	Y	Y	Y	Y	Y	Y	Y	Y	Y	Y	Y	Y	Y	Y	Y	Y	Y	Y	Y	Y	Y	Y	Y	Y	Y	Y	Y	Y	Y	Y	Y	Y	Y	Y	Y	Y	Y	Y	Y	Y	Y	Y	Y	Y
S2. Do the collected data allow to address the research questions?	Y	Y	Y	Y	Y	Y	Y	Y	Y	Y	Y	Y	Y	Y	Y	Y	Y	Y	Y	Y	Y	Y	Y	Y	Y	Y	Y	Y	Y	Y	Y	Y	Y	Y	Y	Y	Y	Y	Y	Y	Y	Y	Y	Y	Y	Y
3.1. Are the participants representative of the target population?	Y	CT	Y	Y	Y	Y	Y	Y	Y	Y	Y	Y	Y	CT	Y	CT	Y	Y	CT	Y	CT	CT	Y	CT	CT	Y	Y	Y	Y	Y	Y	Y	Y	Y	Y	CT	N	Y	Y	Y	Y	CT	Y	Y	CT	CT
3.2. Are measurements appropriate regarding both the outcome and intervention (or exposure)?	Y	Y	Y	Y	Y	Y	Y	Y	Y	Y	Y	Y	Y	Y	Y	Y	Y	Y	Y	Y	Y	Y	Y	Y	Y	Y	Y	Y	Y	Y	Y	Y	Y	Y	Y	Y	Y	Y	Y	Y	Y	Y	Y	Y	Y	Y
3.3. Are there complete outcome data?	Y	Y	Y	Y	Y	Y	Y	Y	Y	Y	Y	Y	Y	Y	Y	Y	Y	Y	Y	Y	Y	Y	Y	Y	Y	Y	Y	Y	Y	Y	Y	Y	Y	Y	Y	Y	Y	Y	Y	Y	Y	Y	Y	Y	Y	Y
3.4. Are the confounders accounted for in the design and analysis?	Y	Y	N	Y	Y	N	Y	Y	Y	Y	Y	Y	Y	Y	Y	Y	N	Y	Y	Y	Y	Y	Y	Y	Y	Y	Y	Y	Y	Y	Y	Y	Y	Y	Y	Y	Y	Y	Y	Y	Y	Y	Y	Y	Y	Y

3.5. During the study period, is the intervention administered (or exposure occurred) as intended?	Y	Y	Y	Y	Y	Y	Y	Y	Y	Y	Y	Y	Y	Y	Y	Y	Y	Y	Y	Y	Y	Y	Y	Y	Y	Y	Y	Y	Y	Y	Y	Y	Y	Y	Y	Y	Y	Y	Y	Y	Y	Y	Y	Y	Y	Y
Total number of criteria met by study	5	4	4	5	5	4	5	5	5	5	5	5	5	4	5	4	4	5	4	5	4	4	5	4	4	5	5	5	5	5	5	5	5	5	5	4	4	5	5	5	5	4	5	5	4	4

Y: Yes, N: No, CT: Can't tell

1. Lu et al., 2021
2. Nicholls et al., 2021
3. Azambuja et al., 2022
4. Che et al., 2022
5. Lovier et al., 2022
6. Maor et al., 2022
7. Pakalniskiene et al., 2022
8. Pietraszek et al., 2022
9. Sulis et al., 2022
10. Vogelsang et al., 2022
11. García-Hernández et al., 2023
12. Liang et al., 2023
13. Luchesi et al., 2023
14. Pestarino et al., 2023
15. Tan et al., 2023
16. You et al., 2023
17. Abline et al., 2024
18. Fuller et al., 2024
19. Huseth-Zosel et al., 2024
20. Niu et al., 2024
21. Riad et al., 2024

22. Sun et al., 2024
23. Ye et al., 2024
24. Zhang et al., 2024
25. Zhao et al., 2024
26. Abu-Helalah et al., 2025
27. Balmuth et al., 2025
28. de et al., 2025
29. Guo et al., 2025
30. Kaneko et al., 2025
31. Kassim et al., 2025
32. Kim et al., 2025
33. Liang et al., 2025
34. Liu et al., 2025
35. Machida et al., 2025
36. Mousa et al., 2025
37. Nuss et al., 2025
38. Rezaei et al., 2025
39. Su et al., 2025
40. Tao et al., 2025
41. Trivedi et al., 2025
42. Wang et al., 2025
43. Yang et al., 2025
44. Yuan et al., 2025
45. Zhao Y et al., 2025
46. Huang et al., 2026

Table S3. Quality assessments of included qualitative studies (assessed by MMAT version 2018).

MMAT Question- Qualitative studies	Baxter et al., 2025	Zhao Q. et al., 2025
S1. Are there clear research questions?	Y	Y
S2. Do the collected data allow to address the research questions?	Y	Y

1.1. Is the qualitative approach appropriate to answer the research question?	Y	Y
1.2. Are the qualitative data collection methods adequate to address the research question?	Y	Y
1.3. Are the findings adequately derived from the data?	Y	Y
1.4. Is the interpretation of results sufficiently substantiated by data?	Y	Y
1.5. Is there coherence between qualitative data sources, collection, analysis and interpretation	Y	Y
Total number of criteria met by study	5	5

Y: Yes, N: No, CT: Can't tell

Table S4. Quality assessments of included mixed methods studies (assessed by MMAT version 2018).

MMAT Question-Mixed methods studies	Waite et al., 2021	Music et al., 2023	Xu et al., 2023
S1. Are there clear research questions?	Y	Y	Y
S2. Do the collected data allow to address the research questions?	Y	Y	Y
5.1. Is there an adequate rationale for using a mixed methods design to address the research question?	CT	Y	Y
5.2. Are the different components of the study effectively integrated to answer the research question?	Y	Y	Y
5.3. Are the outputs of the integration of qualitative and quantitative components adequately interpreted?	Y	Y	Y
5.4. Are divergences and inconsistencies between quantitative and qualitative results adequately addressed?	Y	Y	Y
5.5. Do the different components of the study adhere to the quality criteria of each tradition of the methods involved?	Y	N	Y
Total number of criteria met by study	4	4	5

Y: Yes, N: No, CT: Can't tell

Supplement S3. Determinants of SIV intention in quantitative and qualitative studies.

Socio-demographics and health conditions

Most studies found that higher income [1-3], higher educational level [1, 2], being married [2], being a local resident [4], and living in urban areas [1] were facilitators of SIV intention, whereas being male [1, 4-6] and being employed [1, 5, 6] were identified as barriers, with one study reporting an exception [7]. The role of older age in SIV intention remained mixed, with some studies identifying it as a facilitator [1, 5] and others as a barrier [6, 7]. Unlike SIV uptake, living in nursing homes emerged as a barrier to SIV intention [1], while having a religious belief appeared as a new facilitating factor [2]. Having chronic diseases was widely identified as a facilitator of SIV intention [1, 3, 4, 6], and other study also suggested that a history of vaccine-preventable diseases acted as a facilitator [1, 3, 4, 6]. However, Wang et al. argue that a history of influenza-like illnesses constitutes a barrier, as do depression and the use of influenza-specific medications [2] (**Table S1**).

Lifestyle, history of vaccination, and social media influence

Regarding general health status, some studies suggested that better health status was associated with higher SIV intention [2, 3], whereas others reported that poorer health status was associated with higher intention [5]. Previous history of SIV was a significant facilitator of SIV intention [2, 5, 8, 9], and history of pneumonia vaccination [2, 5] and regular medical checkups [2, 9] were also associated with higher intention to receive SIV. Additionally, Wang et al. noted that thoughtful consideration of the veracity of vaccine-specific information was a facilitator, while overreliance on non-pharmaceutical interventions was a barrier [2].

Knowledge and attitudes toward SIV

Six quantitative studies have consistently found that higher SIV knowledge was associated with higher SIV intention [2, 4, 6-9]. Similar to SIV uptake, perceived vaccine effectiveness was identified as a facilitator of SIV intention [2, 5, 6, 10, 11]. However, no study reported an association between perceiving vaccination as a social responsibility and SIV intention. The most commonly reported perceived barriers to SIV intention were concerns about vaccine safety [2, 5, 11, 12] and perceived inconvenience of vaccination [2, 4, 7, 12], followed by concerns about vaccine affordability [4, 11] and perceived low priority [5, 6], and last, fear of needles [2]. Disease perception, including the perceived susceptibility [7, 9, 11] to and severity of influenza [2, 5], as well as general positive attitudes toward vaccination [8] and trust in healthcare professionals [7], also served as a facilitator of SIV intention. The qualitative study in Canada revealed some specific reasons: older adults believed that they are more susceptible to influenza due to advanced age, and that SIV can effectively protect them from infection, which increased their willingness to receive SIV [13]. Cues to action also play a significant role in SIV intention, with six studies finding that older adults who had received recommendations from health professionals, family members, or the

community exhibited higher SIV intention [2, 3, 5, 6, 11, 12].

COVID-19 related factors

Zhang et al. found that individuals with history of COVID-19 vaccination and those with positive attitudes toward COVID-19 vaccines were more likely to express intention to receive the SIV [10]. Liang et al. found that participants concerned that SIV and COVID-19 vaccination might negatively affect each other's effectiveness expressed a lower intention to receive SIV, whereas those who perceived a higher risk of co-infection with COVID-19 and SIV were more willing to receive SIV [5]. Waite et al. also supplemented more COVID-19-related reasons that make older adults willing to receive SIV through qualitative data: some hoped for cross-protection between influenza and COVID-19, some believed that SIV could reduce the risk of COVID-19 infection, and others felt that COVID-19 had made them more susceptible to infection [13].

Table S5. Determinants of SIV intention in quantitative and qualitative studies determinants of SIV intention in all studies (n=13).

		1	2	3	4	5	6	7	8	9	10	11	12	13
Socio-demographics	Male	🔴		🔴			🔴					🔴		
	Older age		🔴	🟢			🟢					🔴		
	Local residents	🟢												
	Living in urban area		🔴				🟢							
	Living at nursing home/ sanatorium						🔴							
	Higher income						🟢		🟢		🟢			
	Higher education		🔴				🟢		🟢					
	Married								🟢					
	Living with family								🟢					
	Employment			🔴			🔴					🔴		
	Having religious belief								🟢					
Health conditions	General health status			🔴					🟢		🟢			
	Having chronic disease	🟢					🟢				🟢	🟢		
	History of vaccine-preventable diseases	🟢							🔴					
	Having mental health issues								🔴					
	Receiving medication regularly								🔴					
Lifestyles	Regular medical checkup								🟢				🟢	
History of vaccination	Pneumonia vaccination			🟢					🟢					
	SIV			🟢		🟢			🟢			🟢	🟢	
Knowledge and attitudes	General positive attitudes towards vaccination					🟢								
	Perceived disease susceptibility		🟢					🟢	🟢				🟢	🟢
	Perceived disease severity			🟢					🟢					

	Perceived vaccine effectiveness			🟢	🟢			🟢	🟢			🟢		🟢
	Concern of vaccine safety			🔴				🔴	🔴	🔴				
	Concerns of vaccine affordability	🔴						🔴						
	Perceived inconvenience of vaccination	🔴	🔴						🔴	🔴				
	Fear of needles								🔴					
	Perceived low priority			🔴								🔴		
	Higher level of vaccine knowledge	🟢	🟢			🟢			🟢			🟢	🟢	🟢
	Trust to health professional		🟢											
	Received health professional/family/community recommendation			🟢				🟢	🟢	🟢	🟢	🟢		
Factors that are relevant to COVID-19	History of COVID-19 vaccination				🟢				🟢					
	Positive attitude towards COVID-19 vaccines				🟢									
	Concern that SIV and COVID-19 vaccination would negatively affect effectiveness of each other			🔴										
	Perceived higher risk of co-infection with COVID-19 and SIV			🟢										
	Hope for cross-protection between influenza and COVID-19													🟢
	Perceived SIV can reduce COVID-19 vulnerability													🟢
	Perceived vulnerability to severe illness due to COVID-19													🟢
	Perceiving COVID-19 to have a higher infectivity than SIV			🟢										
Social influence	Overreliance on non-pharmaceutical interventions								🔴					

	Thoughtful consideration of the veracity of vaccine-specific information								●					

●Facilitator, ●Barriers.

1. Lu et al., 2021
2. Che et al., 2022
3. Liang et al., 2023
4. Zhang et al., 2024
5. Kassim et al., 2025
6. Liu et al., 2025
7. Su et al., 2025
8. Wang et al., 2025
9. Yang et al., 2025
10. Yuan et al., 2025
11. Zhao Y. et al., 2025
12. Huang et al., 2026
13. Waite et al., 2021

1-12: Quantitative studies and quantitative data in mixed methods studies; 13: qualitative data in mixed methods studies.

Supplement S4. Subgroup analyses of determinants of SIV uptake in quantitative studies.

Table S6. Determinants of SIV uptake by age group.

		Age group												
		50-64	65+											
		1a	1b	2b	3b	4b	5b	6b	7b	8b	9b	10b	11b	12b
Socio-demographics	Male											🔴		
	Older age								🟢			🟢		
	Living in urban area									🔴	🟢			
	Higher income			🟢			🟢			🟢				🟢
	Higher education								🟢	🟢	🟢			🟢
	Married	🟢												
	Living with family						🔴	🔴			🟢	🔴		
	Employment									🔴		🔴		
	Health insurance coverage				🟢					🟢				
Health conditions	General health status			🔴					🔴			🔴		
	Having chronic disease (e.g., cardiovascular disease, hyperlipidemia)	🟢			🟢		🟢					🟢		
	History of vaccine-preventable diseases (e.g.influenza, pneumonia)									🟢				
	Having mental health issues (e.g. anxiety, depression)			🟢										
	History of hospitalization due to influenza									🟢				
Lifestyles	Regular medical checkup			🟢	🟢									

	Smoking	🔴		🔴							🟢			
	Alcohol drinking	🔴										🟢		
	Wearing masks										🟢			
History of vaccination	Pneumonia vaccination			🟢								🟢		
Knowledge and attitudes	General positive attitudes towards vaccination									🟢				
	Perceived disease susceptibility		🟢					🟢		🟢		🟢		
	Perceived disease severity											🟢		
	Perceived vaccine effectiveness					🟢		🟢				🟢		
	Concern of vaccine safety		🔴			🔴		🔴		🔴		🔴		
	Concerns of vaccine affordability											🔴		
	Perceived inconvenience of vaccination											🔴	🔴	
	Fear of needles								🔴					
	Perceived self-efficacy											🟢		
	Higher level of vaccine knowledge							🟢		🟢				
	Perceived vaccination as a social responsibility		🟢			🟢		🟢						
	Trust to health professionals					🟢				🟢				
	Received health professional/family/community recommendation							🟢	🟢	🟢		🟢		
Factors that are relevant to COVID-20	History of COVID-19 vaccination	🟢												
	Perceived high risk of co-infection of SIV and COVID-19											🟢		

	Concerns about the potential interactions of SIV and COVID-19 vaccination											●		

●Facilitator, ●Barriers.

1a Kim et al., 2025

1b Nicholls et al., 2021

2b Lovier et al., 2022

3b García-Hernández et al., 2023

4b Music et al., 2023

5b Tan et al., 2023

6b Xu et al., 2023

7b Fuller et al., 2024

8b Abu-Helalah et al., 2025

9b de et al., 2025

10b Liang et al., 2025

11b Machida et al., 2025

12b Tao et al., 2025

Table S7. Determinants of SIV uptake by vaccination policy context.

		Policy context																									
		**Fully-subsidized**															**Self-financed/partially subsidized**										
		1a	2a	3a	4a	5a	6a	7a	8a	9a	10a	11a	12a	13a	14a	15a	1b	2b	3b	4b	5b	6b	7b	8b	9b	10b	11b
Socio-demographics	Male												🔴				🔴								🔴		
	Older age				🟢		🟢		🟢	🟢	🟢		🟢			🟢					🔴				🟢		
	Local residents															🟢	🟢										
	Living in urban area										🟢	🟢										🔴			🟢		
	Living at nursing home/ sanatorium																								🟢		
	Higher income		🟢		🟢			🟢	🟢		🟢			🟢		🟢		🟢		🟢	🟢	🟢			🟢		
	Higher education				🟢					🟢	🟢	🟢		🟢						🟢		🟢			🟢		
	Married															🟢								🟢			
	Living with family							🔴				🟢	🔴						🔴	🟢							
	Employment												🔴			🔴						🔴					
	Health insurance coverage					🟢										🟢						🟢					
Health conditions	General health status		🔴							🔴	🔴		🔴			🟢		🔴		🔴							
	Having chronic disease (e.g., cardiovascular disease, hyperlipidemia)				🟢	🟢		🟢					🟢				🟢			🟢				🟢			
	History of vaccine-															🟢	🟢				🟢	🟢					

	preventable diseases (e.g.influenza, pneumonia)																										
	Having mental health issues (e.g. anxiety, depression)		🟢																								
	History of hospitalization due to influenza																				🟢	🟢					
Lifestyles	Regular medical checkup		🟢			🟢										🟢											
	Smoking		🔴									🟢				🔴								🔴			
	Alcohol drinking												🟢											🔴			
	Exercise															🟢											
	Wearing masks						🟢					🟢															
History of vaccination	Pneumonia vaccination		🟢										🟢			🟢											
	SIV				🟢																		🟢				
Knowledge and attitudes	General positive attitudes towards vaccination			🟢																	🟢	🟢	🟢				
	Perceived disease susceptibility	🟢											🟢			🟢			🟢	🟢	🟢	🟢				🟢	🟢
	Perceived disease severity												🟢		🟢											🟢	

	Perceived vaccine effectiveness												🟢			🟢			🟢	🟢						🟢	
	Concern of vaccine safety	🔴											🔴			🔴			🔴	🔴		🔴				🔴	🔴
	Concerns of vaccine affordability												🔴			🔴	🔴			🔴	🔴					🔴	🔴
	Perceived inconvenience of vaccination												🔴				🔴									🔴	🔴
	Fear of needles									🔴																	🔴
	Perceived low priority								🔴																		
	Perceived self-efficacy												🟢													🟢	
	Higher level of vaccine knowledge								🟢							🟢			🟢	🟢	🟢	🟢	🟢			🟢	🟢
	Perceived vaccination as a social responsibility	🟢																	🟢							🟢	
	Trust to health professionals																					🟢				🟢	
	Received health professional/family/community recommendation						🟢			🟢			🟢		🟢	🟢			🟢			🟢				🟢	🟢
Factors that are relevant to COVID-20	History of COVID-19 infection															🟢											
	History of COVID-19								🟢							🟢								🟢			

	vaccination																										
	Concerns about COVID-19				🟢																						
	Negative experience of COVID-19				🟢																						
	Perceived high risk of co-infection of SIV and COVID-19												🟢														
	Concerns about the potential interactions of SIV and COVID-19 vaccination												🔴														
Social influence	Thoughtful consideration of the veracity of vaccine-specific information			🟢																							
	Positive experience of healthcare service																									🟢	

🟢Facilitator, 🔴Barriers.

1a Nicholls et al., 2021

2a Lovier et al., 2022

3a Pakalniskiene et al., 2022

4a Sulis et al., 2022

5a García-Hernández et al., 2023

6a Luchesi et al., 2023

7a Tan et al., 2023

8a You et al., 2023
9a Fuller et al., 2024
10a Balmuth et al., 2025
11a de et al., 2025
12a Liang et al., 2025
13a Tao et al., 2025
14a Su et al., 2025
15a Huang et al., 2026
1b Lu et al.
2b Pietraszek et al., 2022
3b Xu et al., 2023
4b Sun et al., 2024
5b Zhao et al., 2024
6b Abu-Helalah et al., 2025
7b Kassim et al., 2025
8b Kim et al., 2025
9b Liu et al., 2025
10b Mousa et al., 2025
11b Su et al., 2025

Table S8. Determinants of SIV uptake by vaccination season.

		Vaccination season																																			
		**During the COVID-19 pandemic**																							**After the COVID-19 pandemic**												
		1a	2a	3a	4a	5a	6a	7a	8a	9a	10a	11a	12a	13a	14a	15a	16a	17a	18a	19a	20a	21a	22a	23a	1b	2b	3b	4b	5b	6b	7b	8b	9b	10b	11b	12b	13b
Socio-demographics	Male	🔴						🔴														🔴	🔴			🔴					🔴						
	Older age						🟢	🟢		🟢		🟢			🟢	🟢						🟢	🟢			🟢	🔴	🟢			🟢						🟢
	Local residents	🟢																								🟢											🟢
	Living in urban area																	🔴	🟢				🟢			🟢		🟢									
	Living at nursing home/ sanatorium																						🟢														
	Higher income			🟢		🟢	🟢	🟢					🟢		🟢		🟢	🟢					🟢	🟢			🟢	🟢	🟢					🟢		🟢	🟢
	Higher education						🟢									🟢	🟢	🟢	🟢				🟢	🟢				🟢	🟢					🟢			
	Married							🟢													🟢								🟢					🟢			🟢
	Living with family							🔴					🔴	🔴			🟢		🟢			🔴															
	Employment							🔴										🔴				🔴									🔴			🔴			🔴
	Health insurance coverage								🟢									🟢							🟢	🟢			🟢								🟢
Health conditions	General health status			🔴		🔴		🔴								🔴	🔴					🔴						🔴	🟢		🔴			🟢		🔴	🟢
	Having chronic disease (e.g., cardiovascular	🟢					🟢		🟢			🟢	🟢				🟢				🟢	🟢			🟢				🟢		🟢			🟢			

	disease, hyperlipidemia)																																				
	History of vaccine-preventable diseases (e.g.influenza, pneumonia)	🟢																🟢								🟢	🟢										🟢
	Having mental health issues (e.g. anxiety, depression)			🟢																																	
	History of hospitalization due to influenza																	🟢									🟢										
	Receiving medication regularly																								🟢												
Lifestyles	Regular medical checkup			🟢				🟢	🟢																												🟢
	Smoking			🔴				🔴											🟢		🔴																🔴
	Alcohol drinking							🟢													🔴										🟢						
	Exercise																																				🟢
	Wearing seat belt							🟢																													
	Wearing masks									🟢									🟢																		
History of vaccination	Pneumonia vaccination			🟢																		🟢			🟢						🟢						🟢
	SIV						🟢																			🟢				🟢				🟢			

Knowledge and attitudes	General positive attitudes towards vaccination				🟢													🟢							🟢		🟢			🟢							
	Perceived disease susceptibility		🟢											🟢			🟢	🟢							🟢		🟢		🟢		🟢		🟢		🟢		🟢
	Perceived disease severity																					🟢									🟢		🟢		🟢		
	Perceived vaccine effectiveness										🟢			🟢			🟢					🟢			🟢				🟢		🟢		🟢				🟢
	Concern of vaccine safety		🔴								🔴			🔴			🔴	🔴				🔴			🔴				🔴		🔴		🔴		🔴		🔴
	Concerns of vaccine affordability	🔴															🔴					🔴					🔴				🔴		🔴		🔴		🔴
	Perceived inconvenience of vaccination	🔴																				🔴									🔴	🔴	🔴		🔴		
	Fear of needles															🔴																			🔴		
	Perceived low priority														🔴																						
	Perceived self-efficacy																					🟢							🟢		🟢		🟢				
	Higher level of vaccine knowledge													🟢	🟢		🟢	🟢							🟢		🟢			🟢			🟢		🟢		🟢
	Perceived vaccination as a social responsibility		🟢								🟢			🟢											🟢								🟢				

	Trust to health professionals										🟢							🟢															🟢	🟢			
	Received health professional/family/community recommendation									🟢				🟢		🟢		🟢				🟢							🟢		🟢		🟢	🟢	🟢	🟢	🟢
Factors that are relevant to COVID-20	History of COVID-19 infection																																				🟢
	History of COVID-19 vaccination														🟢						🟢				🟢	🟢								🟢			🟢
	General positive attitude towards COVID-19						🟢																														
	Negative experience of COVID-19						🟢																														
	Perceived high risk of co-infection of SIV and COVID-19																					🟢									🟢						
	Concerns about the potential interactions of SIV and COVID-19 vaccination																					🔴									🔴						

Social influence and trust	Social media promotion																											●									
	Thoughtful consideration of the veracity of vaccine-specific information				●																			●													
	Positive experience of healthcare service																			●												●					

●Facilitator, ●Barriers.

1a Lu et al.

2a Nicholls et al., 2021

3a Lovier et al., 2022

4a Pakalniskiene et al., 2022

5a Pietraszek et al., 2022

6a Sulis et al., 2022

7a Vogelsang et al., 2022

8a García-Hernández et al., 2023

9a Luchesi et al., 2023

10a Music et al., 2023

11a Pestarino et al., 2023

12a Tan et al., 2023

13a Xu et al., 2023

14a You et al., 2023

15a Fuller et al., 2024

16a Sun et al., 2024

17a Abu-Helalah et al., 2025
18a de et al., 2025
19a Kaneko et al., 2025
20a Kim et al., 2025
21a Liang et al., 2025
22a Liu et al., 2025
23a Tao et al., 2025
1b Riad et al., 2024
2b Ye et al., 2024
3b Zhao et al., 2024
4b Balmuth et al., 2025
5b Guo et al., 2025
6b Kassim et al., 2025
7b Liang et al., 2025
8b Machida et al., 2025
9b Mousa et al., 2025
10b Rezaei et al., 2025
11b Su et al., 2025
12b Yuan et al., 2025
13b Huang et al., 2026

Supplement S5. Reasons for SIV uptake in qualitative data.

Table S9. Reasons for SIV uptake in qualitative data (n=10).

		Azambuja et al., 2022	Maor et al., 2022	Music et al., 2023	Xu et al., 2023	Abline et al., 2024	Huseth-Zosel et al., 2024	Baxter et al., 2025	Nuss et al., 2025	Rezaei et al., 2025	Zhao Q. et al., 2025
Socio-demographics	Higher income										🟢
	Health insurance coverage				🟢						
History of vaccination	SIV								🟢		
Knowledge and attitudes	Perceived disease susceptibility				🟢		🟢		🟢	🟢	
	Perceived disease severity					🟢				🟢	
	Perceived vaccine effectiveness		🟢			🟢	🟢		🟢		
	Concern of vaccine safety		🔴	🔴	🔴	🔴	🔴			🔴	
	Concerns of vaccine affordability									🔴	
	Perceived inconvenience of vaccination	🔴						🔴			🔴

	Perceived low priority				🔴						
	Higher level of vaccine knowledge							🟢	🟢	🟢	
	Perceived vaccination as a social responsibility				🟢	🟢		🟢	🟢		
	Trust to health professional	🟢		🟢				🟢			
	Received health professional/family /community recommendation				🟢	🟢			🟢	🟢	🟢
Factors that are relevant to COVID-19	Perceived impact of COVID-19 pandemic on health system reduced	🟢									
	Fear of leaving home due to the COVID-19 pandemic	🔴									
Social influence	Social media promotion	🟢		🟢							

	Positive experience of healthcare service			●				●			

●Facilitator, ●Barriers.

Supplement S6. Subgroup analysis of meta-analysis on SIV uptake.

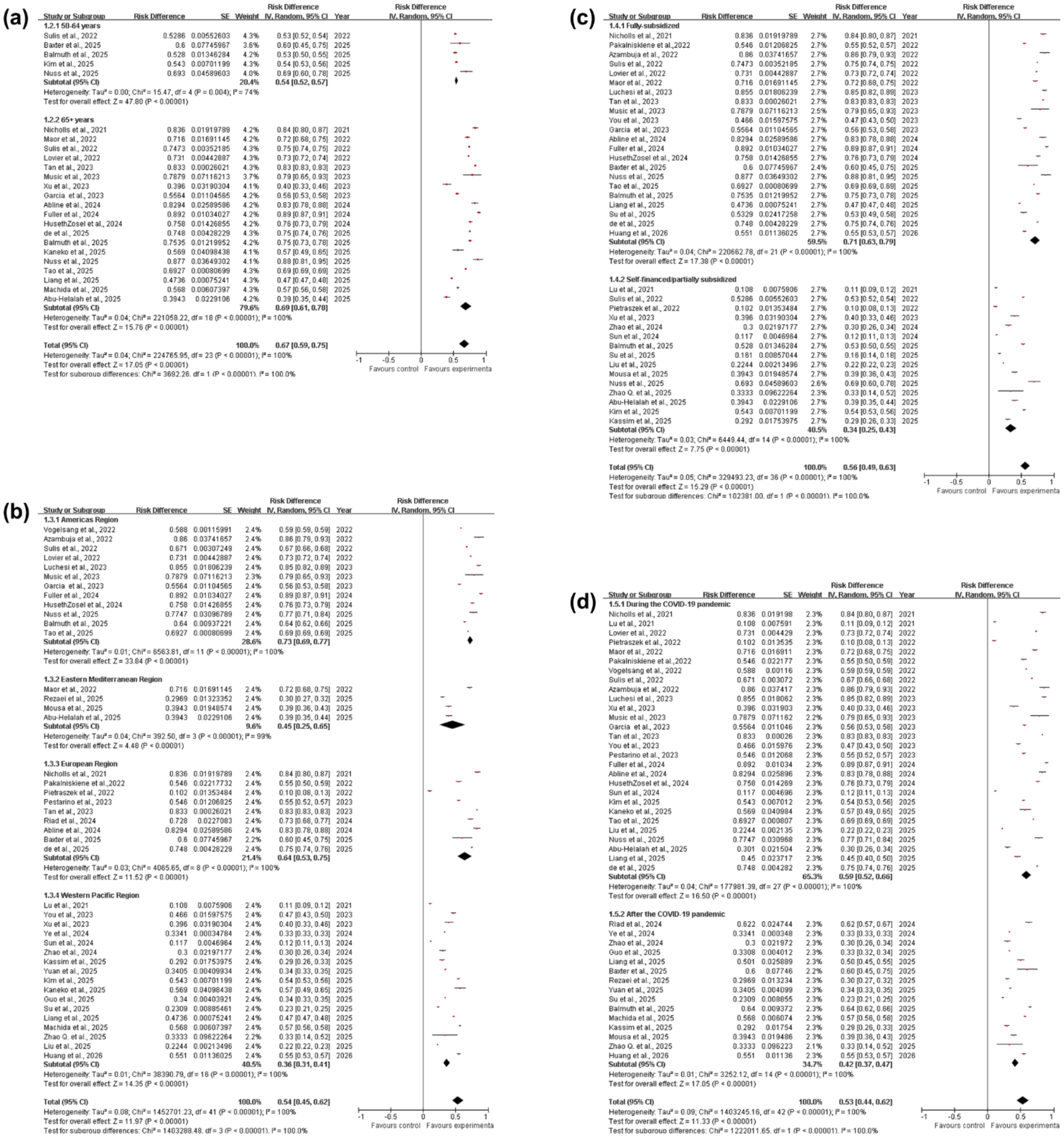

Figure S1. Forest plot for subgroup analysis of pooled prevalence of SIV uptake: (a) age group; (b) region; (c) policy context; (d) vaccination season.

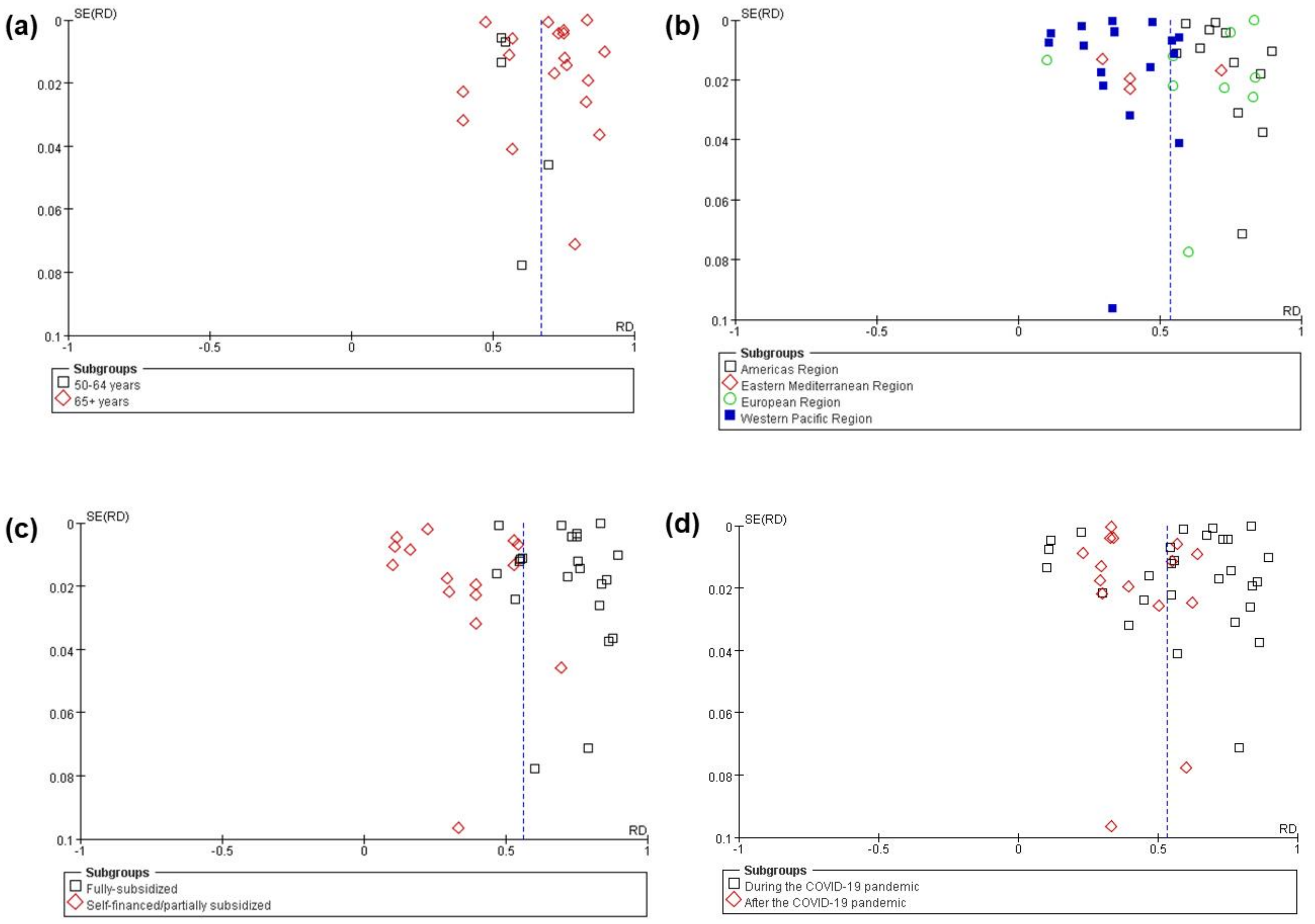

Figure S2. Funnel plot of publication bias for subgroup analyses of SIV uptake (a) age group; (b) region; (c) policy context; (d) vaccination season.

Supplement S7. Meta-analysis on SIV intention and its subgroup analysis.

A total of 13 studies reported SIV intention as an outcome, and 86,756 participants were included. The pooled prevalence of SIV intention was 62% (95% CI: 51–74%). No evidence of publication bias was detected, although substantial heterogeneity was observed across studies (**Table 5, Figure S3, and Figure S4**). Subgroup analysis indicated that the prevalence of SIV intention was higher in settings with fully-subsidized SIV (pooled prevalence = 74%, 95% CI: 68-80%; I^2 = 100%, p < 0.001) than in those with self-financed/partially subsidized vaccination policies (pooled prevalence = 53%, 95% CI: 36-70%; I^2 = 99%, p < 0.001). For vaccination season, SIV intention prevalence during the COVID-19 pandemic (pooled prevalence = 73%, 95% CI: 66-80%; I^2 = 100%, p < 0.001) was also higher than that after the COVID-19 pandemic (pooled prevalence = 56%, 95% CI: 39-73%; I^2 = 100%, p < 0.001). (**Figure S5, and Figure S6**).

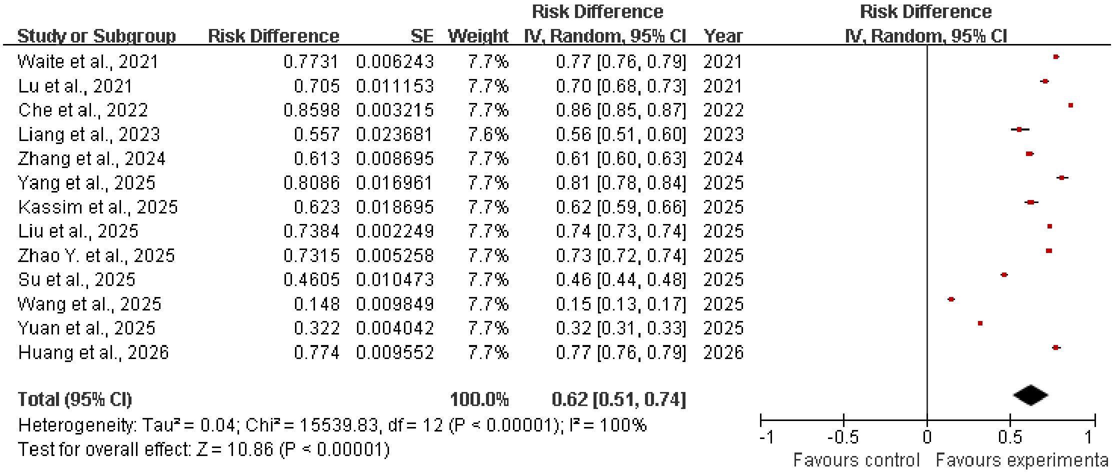

Figure S3. Forest plot of the pooled prevalence for SIV intention.

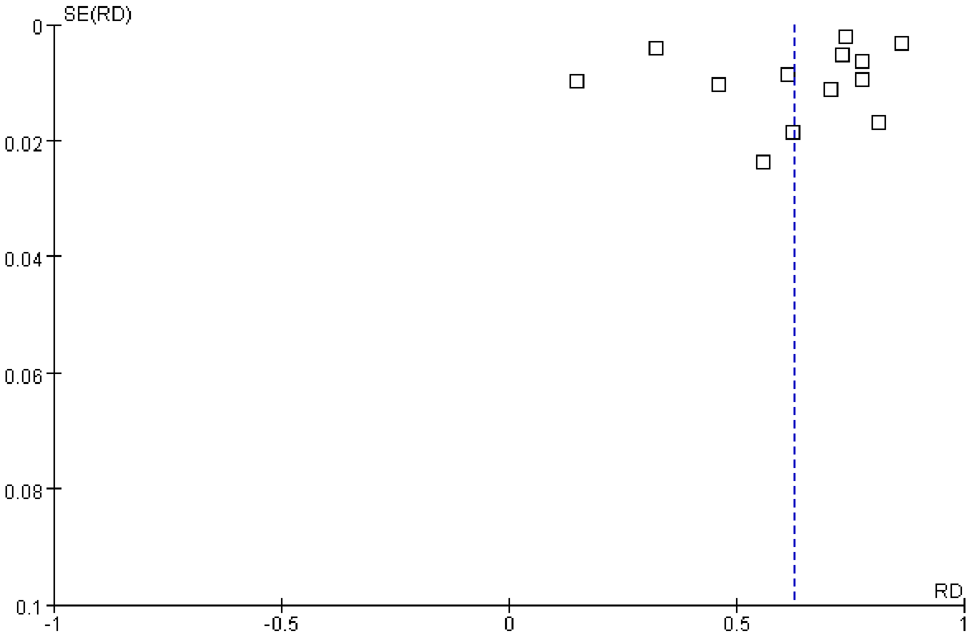

Figure S4. Funnel plot of publication bias among the included studies on SIV intention.

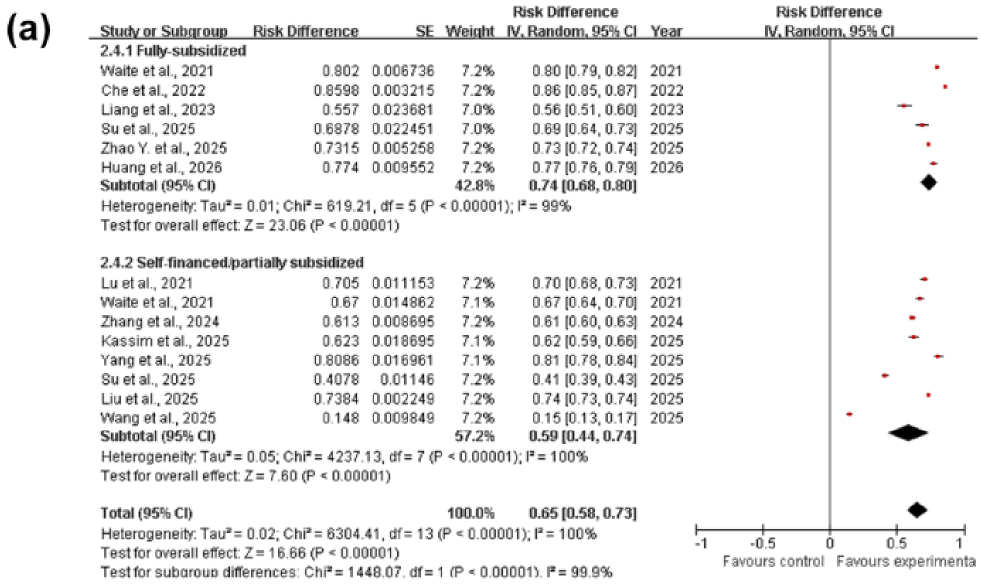

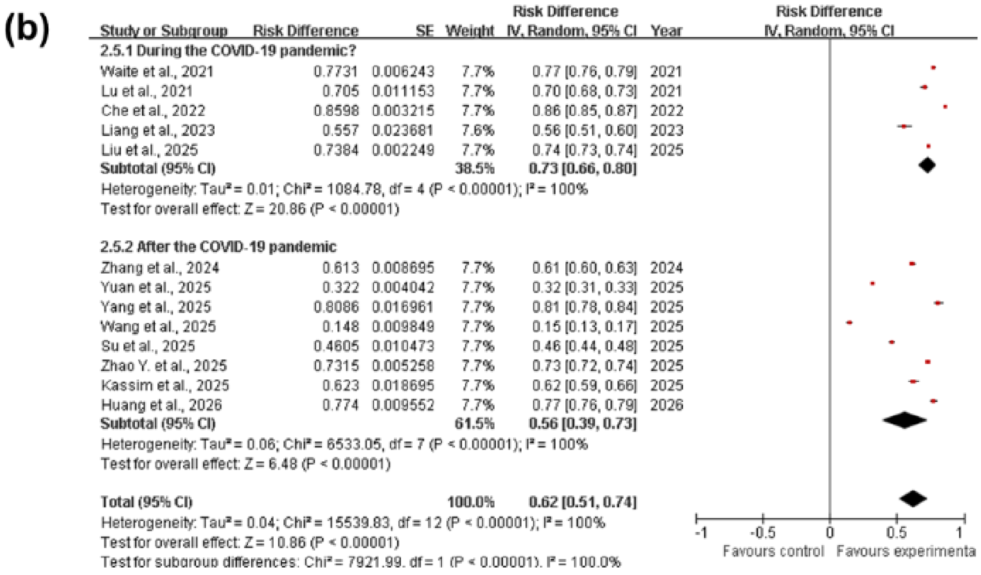

Figure S5. Forest plot for subgroup analysis of pooled prevalence of SIV intention: (a) policy context; (b) vaccination season.

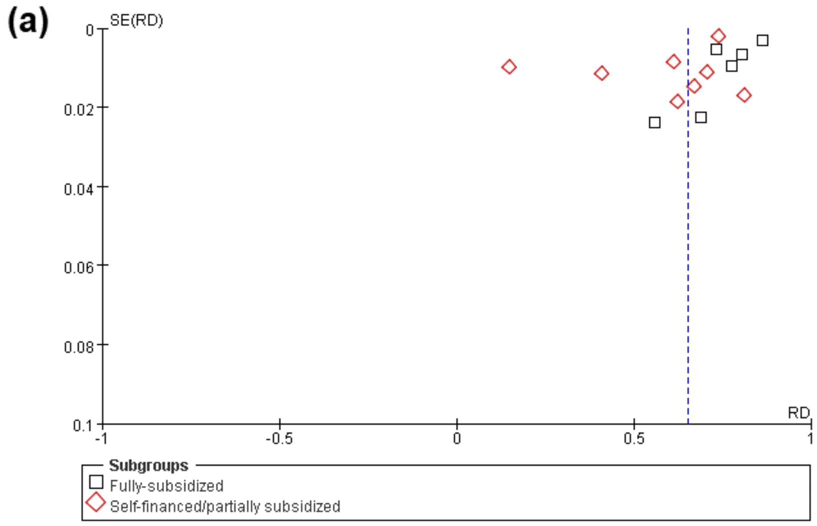

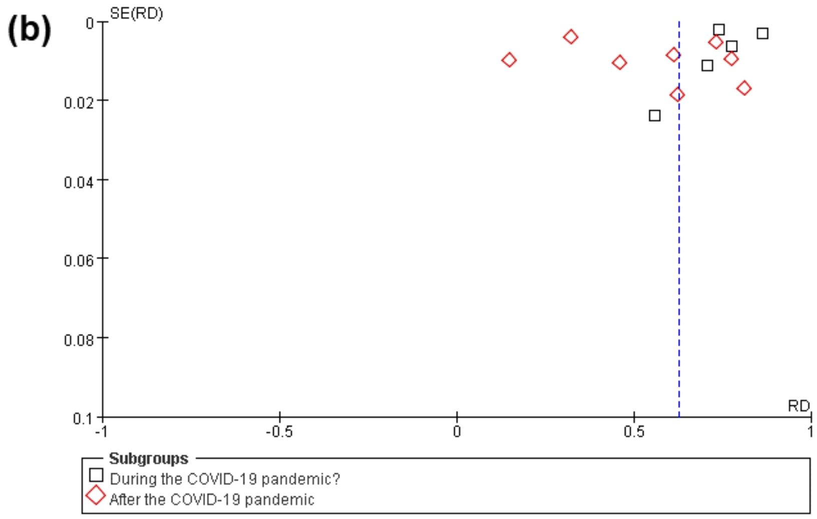

Figure S6. Funnel plot of publication bias for subgroup analyses of SIV intention (a) policy context; (b) vaccination season.

References:

1. Liu, D., et al., *Influenza, pneumococcal, COVID-19 vaccine willingness and uptake with influencing factors in 38,184 chinese older adults in 2022: a nationwide cross-sectional study.* Vaccine, 2025. **68**: p. 127961.
2. Wang, X., et al., *Why do older adults hesitate to get the flu vaccine? A cross-sectional study on vaccine hesitancy in the post-COVID-19 era.* Front Public Health, 2025. **13**: p. 1603091.
3. Yuan, S., et al., *The Mediating Role of Vaccine Hesitancy in Influenza Vaccination Uptake and Intention Among Older Adults in Urban China: Based on a Structural Equation Modeling Study.* VACCINES, 2025. **13**(12).
4. Lu, X., et al., *Gap between willingness and behavior in the vaccination against influenza, pneumonia, and herpes zoster among Chinese aged 50-69 years.* Expert Rev Vaccines, 2021. **20**(9): p. 1147-1152.
5. Liang, X., et al., *Associations between COVID-19 Vaccination and Behavioural Intention to Receive Seasonal Influenza Vaccination among Chinese Older Adults: A Population-Based Random Telephone Survey.* VACCINES, 2023. **11**(7).
6. Zhao, Y., et al., *Impact of a Free Influenza Vaccination Policy on Older Adults in Zhejiang, China: Cross-Sectional Survey of Vaccination Willingness and Determinants.* JMIR Hum Factors, 2025. **12**: p. e73940.
7. Che, X., et al., *Analysis on the intention and influencing factors of free influenza vaccination among the elderly people aged 70 and above in Hangzhou in 2022.* Front Public Health, 2022. **10**: p. 1052500.
8. Kassim, M.S.A., et al., *Challenges in Integrating Influenza Vaccination Among Older People in National Immunisation Program: A Population-Based, Cross-Sectional Study on Knowledge, Attitudes, Practices, and Acceptance of a Free Annual Program.* VACCINES, 2025. **13**(6).
9. Huang, F., et al., *COVID-19 as a catalyst? Uptake and drivers of seasonal influenza and pneumococcal vaccination among older adults in post-pandemic Shenzhen, China.* Vaccine, 2026. **69**: p. 128013-128013.
10. Zhang, S.S., et al., *Impact of propensity to COVID-19 vaccination/vaccine on influenza vaccination from willingness to behavior among older adults in rural China.* Hum Vaccin Immunother, 2024. **20**(1): p. 2428017.
11. Su, R.A., et al., *Influenza Vaccination in the Elderly in Three Cities in China: Current Status and Influencing Factors Under Different Funding Policies.* VACCINES, 2025. **13**(11).
12. Yang, K., S. Jiang, and J. Jiang, *Influencing factors of influenza vaccination willingness among the elderly in Wuxi city: A study based on the Behavioral and Social Drivers (BeSD) framework and structural equation modeling.* Hum Vaccin Immunother, 2025. **21**(1): p. 2559508.
13. Waite, N.M., et al., *COVID-19's Impact on Willingness to Be Vaccinated against Influenza and COVID-19 during the 2020/2021 Season: Results from an Online Survey of Canadian Adults 50 Years and Older.* VACCINES, 2021. **9**(4).
